# Microfabrication Strategies for Silicon Anodes in On-Chip and Miniaturized Batteries

**DOI:** 10.3390/mi17091103

**Published:** 2026-09-21

**Authors:** Heonsu Park, Churl Seung Lee, Joonho Bae

**Affiliations:** 1 Department of Chemistry and Chemical Engineering, Inha University, 100 Inha-ro, Michuhol-gu, Incheon 22212, Republic of Korea; oldwaterpark@inha.ac.kr; 2 ICT Nano Convergence Technology Research Center, Korea Electronics Technology Institute (KETI), Bundang-gu, Seongnam-si 13509, Republic of Korea; 3 Department of Physics and Semiconductor Science, Gachon University, Seongnam-si 13120, Republic of Korea; 4 Department of Semiconductor Engineering, Gachon University, Seongnam-si 13120, Republic of Korea

**Keywords:** silicon anode, microbattery, on-chip battery, miniaturized lithium-ion battery, thin-film battery, 3D microbattery, CMOS-compatible battery, silicon nanowire, solid-state battery

## Abstract

The rapid expansion of autonomous microsystems, implantable sensors, wireless sensor nodes, Internet-of-Things devices, distributed electronics, and heterogeneous system-on-chip platforms has intensified the demand for compact electrochemical energy-storage systems that can be integrated directly with microfabricated devices. Among the various negative electrode materials, silicon is particularly attractive for miniaturized lithium-ion batteries because of its high theoretical lithium-storage capacity, abundance, compatibility with mature semiconductor processing, and direct availability as both an active material and a structural platform. However, the practical implementation of silicon anodes in on-chip and miniaturized batteries remains difficult because lithiation-induced volume expansion, fracture, unstable solid-electrolyte interphase formation, loss of electrical contact, and process-integration constraints become more severe as the battery footprint is reduced to the microscale. In contrast to conventional slurry-cast silicon electrodes, silicon anodes for microbatteries can exploit microfabrication strategies such as thin-film deposition, photolithography, deep reactive ion etching, metal-assisted chemical etching, nanoimprint lithography, laser patterning, template-assisted growth, atomic layer deposition, and wafer-level encapsulation. These methods enable deterministic control over electrode geometry, areal loading, porosity, current-collector contact, diffusion length, mechanical compliance, interfacial chemistry, and compatibility with complementary metal-oxide-semiconductor and microelectromechanical-system platforms. This review summarizes the recent progress in microfabrication strategies for silicon anodes in on-chip and miniaturized batteries, emphasizing the relationship between the process route, electrode architecture, mechanical stability, electrochemical performance, and manufacturability.

## 1. Introduction

This review focuses specifically on the intersection between silicon-anode chemistry and microfabrication rather than on silicon powder electrodes alone. This distinction is important because wafer-level microbatteries introduce design variables that are largely absent from conventional composite electrodes, including photomask layout, etch anisotropy, sidewall roughness, solid-electrolyte conformality, packaging volume, and process compatibility. The objective is therefore not to rank silicon structures by surface area alone, but to evaluate how deterministic fabrication choices translate into electrochemical performance and integrable device architectures [1,2,3,4].

The organization of this review follows the process-structure-performance logic. First, the design constraints of footprint-limited silicon anodes are discussed in terms of areal capacity, stress evolution, transport, and CMOS/MEMS compatibility. Subsequent sections examine planar films, patterned islands, etched three-dimensional silicon structures, silicon nanowires, porous silicon, coatings, current collectors, and full-cell integration. For each family of approaches, emphasis is placed on how the microfabrication route determines the morphology and failure mode, how the morphology affects electrochemical behavior, and how the resulting electrode can or cannot be integrated into a practical miniaturized battery. This framework is intended to help compare planar thin-film cells, 3D all-solid-state microbatteries, printed hybrid microcells, and wafer-compatible silicon nanostructure electrodes on a common basis [3,4,5,6,7,8,9,10,11,12,13,14,15,16,17,18,19,20,21,22,23,24,25,26,27].

The literature considered in this review was identified primarily through searches of Web of Science, Scopus, and Google Scholar using combinations of keywords such as “silicon anode,” “microbattery,” “on-chip battery,” “3D microbattery,” “thin-film battery,” “silicon nanowire,” “porous silicon,” “microfabrication,” and “CMOS/MEMS integration.” Particular emphasis was placed on studies relevant to microfabricated or miniaturized silicon-anode systems, including device architecture, fabrication process, electrochemical performance, full-cell integration, and manufacturability. Both foundational studies and recent publications were considered, whereas studies focused exclusively on conventional slurry-cast silicon powder electrodes without clear relevance to microscale fabrication or device integration were generally excluded. Because this article is intended as a critical narrative review rather than a systematic review or meta-analysis, the literature set was selected for relevance to the process–structure–performance–integration framework rather than through a formal systematic-screening protocol.

The miniaturization of electronic systems has created a growing mismatch between the scaling of functional devices and that of their power sources [1,2,28,29]. Sensors, actuators, radio-frequency identification tags, implantable biomedical devices, microscale robots, environmental monitors, and heterogeneous integrated circuits continue to shrink in size and increase in functional density, whereas the batteries that power them often remain externally mounted, packaged separately, or poorly matched to the device footprint [1,2,28,29,30,31]. This mismatch has stimulated research on miniaturized batteries and on-chip energy-storage systems that can be fabricated directly on silicon wafers, flexible substrates, or microelectromechanical-system platforms [1,2,5,6,7,8,9,29,30,31,32,33].

Microscale batteries differ fundamentally from conventional coin, pouch, or cylindrical cells because their available footprint is limited, inactive components occupy a larger fraction of the total device volume, and ionic transport distances must be carefully controlled [2,28,29,32,33]. In conventional batteries, the capacity can be increased by thickening the electrodes or stacking a large number of layers; however, electrode thickening can severely degrade rate capability, mechanical reliability, and integration yield [1,2,5,6,7,8,29,30,32,33]. The most relevant performance metrics are areal capacity, volumetric energy density, footprint-normalized power density, mechanical robustness, packaging volume, process compatibility, and system-level deliverable energy [1,2,5,6,7,8,9,30,31,32,33].

Silicon is one of the most promising anode materials for lithium-ion batteries because it can alloy with lithium at a theoretical gravimetric capacity far exceeding that of graphite [34,35,36,37,38,39,40,41,42]. In the context of on-chip and miniaturized batteries, silicon has an additional and distinctive advantage: it is not merely an active battery material but also the dominant substrate and structural material of the semiconductor and MEMS industries [1,2,5,6,7,8,9,30,31,32,33]. A silicon wafer can simultaneously serve as a mechanical support, a microfabrication platform, a current pathway after suitable doping or metallization, and an electrochemically active lithium-storage medium [2,5,6,7,8,9,30].

The properties that make silicon attractive also make it difficult to implement in practical cells [37,38,39,40,41,42,43,44,45]. During lithiation and delithiation, silicon undergoes large volume changes that can generate cracking, pulverization, delamination from current collectors, repeated rupture and reformation of the solid-electrolyte interphase, irreversible lithium loss, and progressive impedance growth [37,38,39,40,41,42,43,44,45,46,47,48,49,50]. These degradation modes are well known in bulk silicon anode research, but they are especially critical in microbatteries because the small cell footprint leaves little tolerance for local failure, nonuniform wetting, imperfect encapsulation, or loss of contact in a small fraction of the active area [1,2,5,6,7,8,9,30,31,32,33].

Microfabrication provides a route to address these difficulties by engineering the shape, size, spacing, porosity, coating, and integration sequence of silicon anodes [1,2,4,5,6,7,8,9,12,13,14,15,16,32,33]. Silicon can be made sufficiently thin to reduce stress, patterned into isolated islands to suppress long crack propagation, etched into high-aspect-ratio structures to increase areal loading, converted into nanowires or porous silicon to create free volume, and conformally coated with artificial interphases or solid electrolytes to stabilize the interface [4,5,6,7,8,9,12,13,14,15,16,51,52,53,54,55,56,57,58,59,60,61]. Therefore, the objective of this review is to analyze silicon anodes as microfabricated active structural components rather than only as high-capacity powder materials.

**Figure 1:**
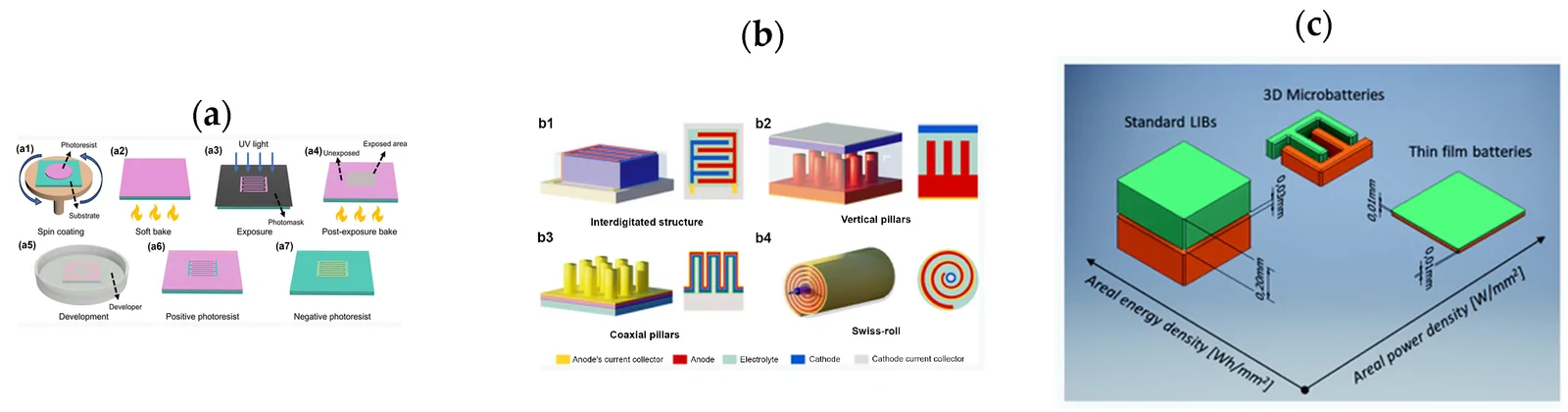
Foundational patterning and architecture concepts for silicon-anode microbatteries. (**a**) Photolithography sequence showing spin coating, soft bake, mask exposure, development, and positive/negative photoresist pattern formation [1]. (**b**) Representative 3D Si micro-LIB architectures, including interdigitated, vertical-pillar, coaxial, and Swiss-roll layouts [2]. (**c**) Comparison of standard LIBs, 3D microbatteries, and thin-film batteries in terms of areal energy- and power-density considerations [30]. Panels (**a**,**b**) are adapted from Refs. [1,2] under the Creative Commons Attribution 4.0 International License; panel (**c**) is adapted from Ref. [30] under the Creative Commons Attribution-NonCommercial 3.0 Unported Licence. Within panel (**a**), the internal source-image labels a–g denote spin coating, soft bake, exposure, post-exposure bake, development, positive-photoresist pattern formation, and negative-photoresist pattern formation, respectively; for unambiguous reference in this caption, these are designated (**a1**)–(**a7**). Within panel (**b**), the internal source-image labels a–d denote interdigitated, vertical-pillar, coaxial-pillar, and Swiss-roll architectures, respectively, and are designated (**b1**)–(**b4**). These caption designations distinguish the reproduced source-internal labels from the top-level panels (**a**–**c**).

In Figure 1a, the basic photolithography sequence is highlighted as the starting point for translating silicon-anode designs into wafer-level microbattery patterns. Figure 1b summarizes how the same lithographic logic is used to form interdigitated current collectors.

## 2. Design Requirements for Silicon Anodes in On-Chip and Miniaturized Batteries

### 2.1. Footprint-Limited Energy Storage

A useful method for evaluating footprint-limited electrodes involves separating the geometric area from the electrochemically accessible area. Micropillars, trenches, and nanowires increase the true active surface area while leaving the lithographic footprint unchanged. However, this benefit is only valuable when the electrolyte can access the sidewalls and the cathode capacity can be balanced against the added anode capacity. If the anode is overdesigned relative to the cathode, excess silicon may contribute to irreversible lithium loss, parasitic surface reactions, and mechanical instability without increasing the full-cell energy. Consequently, silicon-anode microbattery papers should report not only gravimetric capacity but also areal capacity, footprint-normalized energy density, active silicon volume, inactive-stack volume, and packaged-device volume [1,2,3,5,6,7,8,9,28,29,30,31,32,33,62,63,64].

The target current profile is also important in on-chip systems. A microsensor node may require low average power but short high-current bursts during data acquisition or wireless transmission, whereas an implantable or memory-backup device may prioritize long retention and leakage suppression. These different use cases lead to different electrode geometries: high-surface-area nanowires favor high-rate access but can increase the SEI area, dense micropillars improve areal loading but become harder to wet and coat, and thin films simplify integration but limit capacity. Thus, footprint-limited design is a multi-objective optimization problem in which capacity, rate, lifetime, and process yield must be optimized together, rather than individually [1,2,3,5,6,7,29,30,31,32,33,65,66,67].

For miniaturized systems, the first design constraint is the available footprint [1,2,28,29,30,31]. A microbattery integrated near a sensor, circuit, or MEMS device must store useful energy per unit area while occupying minimal lateral space. This shifts attention from gravimetric capacity alone to areal capacity, volumetric energy density, and packaged-device energy density [1,2,5,6,7,8,9,29,30,31,32,33]. A very thin silicon film may exhibit excellent cycling stability but store insufficient charge per footprint, whereas a thick film may provide a higher areal capacity but suffer from fracture, delamination, and slow lithium transport [37,38,39,40,41,42,43,44,45,46,47,48,49,50].

Three-dimensional silicon architectures address footprint constraints by vertically extending the active electrode above or into the substrate [2,5,6,7,8,9,30,32,33]. Micropillars, microtrenches, nanopillars, nanowires, porous silicon, and interdigitated electrodes increase the electrochemically accessible surface area per unit footprint while preserving short diffusion lengths [4,5,6,7,8,9,12,13,14,15,16,32,33]. However, a high surface area also increases the area available for electrolyte decomposition and solid-electrolyte interphase growth; therefore, the optimum architecture must balance active loading, interfacial stability, and electrolyte accessibility [41,42,43,44,45,56,57,58,59,60,61].

### 2.2. Mechanical Accommodation of Silicon Volume Expansion

The mechanical advantages of microfabricated silicon anodes arise from their geometric control. In a continuous film, lithiation produces substrate-constrained biaxial stress over a large lateral distance, which promotes channel cracking and delamination once the stored elastic energy exceeds the interfacial fracture resistance. Patterned islands reduce the lateral constraint length, nanowires and pillars introduce free sidewalls for radial expansion, and porous or hollow structures provide internal void volume for swelling. These approaches do not eliminate silicon expansion; rather, they redirect expansion into geometrically available space and localize damage such that the entire electrode does not fail at once [16,37,38,39,40,41,42,43,44,45,46,47,48,49,50,68,69,70,71].

The mechanical design also controls interface stability. Repeated expansion and contraction can rupture the solid-electrolyte interphase, expose fresh silicon, and consume lithium and electrolyte in every cycle. Microfabricated structures with sharp corners, scalloped DRIE sidewalls, or nonuniform pillar diameters can concentrate stress and accelerate local SEI rupture. Conversely, rounded sidewalls, conformal coatings, and distributed void spaces can reduce stress gradients. Therefore, post-cycling cross-sectional SEM/TEM imaging and stress or curvature measurements are essential for linking the designed geometry to the actual failure mechanisms. A silicon-anode microbattery should be considered mechanically successful only when the structure retains electrical contact, preserves electrolyte access, and limits continuous SEI growth under realistic depth-of-discharge conditions [3,16,17,18,19,20,21,46,47,48,49,50,68,72,73,74].

The primary silicon failure modes—volume expansion, cracking, SEI rupture, and contact loss—were introduced above and are not repeated in each architecture section. The remainder of this section therefore focuses on how geometry changes the distribution of those failure modes: free sidewalls, isolated islands, pores, and hollow volumes redirect expansion into available space, whereas sharp corners, rough sidewalls, and nonuniform feature dimensions concentrate local stress [46,47,48,49,50].

### 2.3. Ion and Electron Transport

Ion transport in microstructured silicon depends strongly on the spacing between the features. A sparse array allows easy electrolyte penetration but sacrifices the active-material packing density, whereas a dense array increases capacity per footprint but may create tortuous or partially blocked transport pathways. In liquid or gel electrolytes, the wetting of deep trenches and nanowire forests can be limited by capillary effects, trapped gas, and surface chemistry. In all-solid-state cells, the challenge shifts to conformal deposition and interfacial contact. The electrolyte must coat the sidewalls without pinholes, the cathode must fill or cover the same 3D geometry, and the total stack must remain electrically isolated. These requirements explain why the optimum aspect ratio in a practical full cell can be lower than the aspect ratio that maximizes silicon surface area in a half cell [3,4,5,6,7,8,9,10,11,12,13,14,15,16,17,18,19,20,21,22,32,33,75,76,77].

Therefore, the electron transport must be treated with equal care. Highly doped silicon substrates, metal seed layers, silicides, and conductive coatings can provide low-resistance pathways; however, they add inactive volume and may introduce contamination or diffusion concerns. A vertical silicon structure is attractive because each feature is naturally connected to the wafer; however, the local contact resistance between the active silicon, the current collector, and the external pad affects rate capability. In nanowire or porous-silicon electrodes, loss of contact after repeated swelling can create electronically isolated regions, even when silicon remains physically present. Therefore, impedance spectroscopy, rate testing, and cross-sectional current-collector analysis should be combined to distinguish between ionic transport limitations and electronic disconnection [4,5,6,7,8,9,12,13,14,15,16,56,57,58,59,60,61,78,79,80,81,82,83,84].

On-chip systems often require intermittent high-power pulses for sensing, wireless transmission, memory backup, or actuation [1,2,28,29,30,31]. High-rate operation requires short lithium-ion transport pathways, continuous electronic pathways, and low interfacial impedance [5,6,7,8,9,32,33]. Silicon is not sufficiently conductive in its pristine state for all microbattery geometries; therefore, microfabricated silicon anodes often require doped silicon substrates, metal current collectors, silicide contacts, carbon coatings, and conductive scaffolds [4,5,6,7,8,9,12,13,14,15,16,56,57,58,59,60,61,78,79,80,81].

Three-dimensional microbattery layouts can shorten ionic transport distances by interpenetrating anode, electrolyte, and cathode structures [5,6,7,8,9,32,33]. However, increasing the aspect ratio complicates the conformal deposition of solid electrolytes and cathodes because sputtering and evaporation are line-of-sight processes, while atomic layer deposition offers excellent conformality but slow growth rates [3,4,12,13,14,15,16,17,18,19,20,21]. Consequently, the anode design must be co-optimized with the deposition route for the electrolyte, cathode, and encapsulation layers [1,2,3,5,6,7,8,9,17,18,19,20,21,30,31,32,33].

### 2.4. CMOS and MEMS Process Compatibility

CMOS/MEMS compatibility is not a single requirement but a set of constraints that influence every step of the battery process flow. The maximum thermal budget limits cathode crystallization and annealing; contamination rules restrict mobile ions, transition metals, and process chemicals; topography affects lithographic alignment and planarization; and final encapsulation must protect both the battery and the surrounding electronics. Silicon-anode fabrication is relatively compatible because etching, oxidation, doping, and metallization are standard wafer processes. The primary challenge is integrating lithium-containing electrolytes and cathodes, which may require sputtering, ALD, pulsed-laser deposition, sol–gel processing, or low-temperature crystallization routes that are less mature in semiconductor lines [1,2,8,9,22,30,31,58,59,60,61,78,79,80,81,85,86,87].

From a manufacturing perspective, yield and uniformity may be more important than peak half-cell capacity. A wafer-level microbattery must have a consistent electrode height, sidewall profile, coating thickness, and package integrity across many dies. A slight etch nonuniformity can change local current density, nonconformal electrolyte deposition can create shorts, and packaging defects can lead to moisture ingress. These issues are rarely visible in the single best-performing test cell. Therefore, future reports should include wafer-scale statistics, process windows, alignment tolerances, and failure analysis, in addition to electrochemical curves. Such information is essential for judging whether the silicon-anode architecture is a laboratory demonstration or a viable on-chip power source [1,2,3,6,7,8,9,30,31].

For true on-chip integration, the silicon anode process must be compatible with semiconductor and MEMS manufacturing constraints [1,2,5,6,7,8,9,30,31,32,33]. These constraints include low contamination risk, acceptable thermal budget, wafer-level uniformity, compatibility with passivation and interconnect metals, and avoidance of materials that degrade transistor or MEMS operation [1,2,5,6,7,8,9,30,31,32,33]. Silicon patterning is already central to microfabrication, but complete battery integration additionally requires cathode deposition, electrolyte formation, current collectors, packaging, and possibly prelithiation [1,2,5,6,7,8,9,10,11,22,29,30,31,32,33].

Recently, CMOS-compatible lithium-ion microbatteries have been proposed as a route toward distributed on-chip power architectures, emphasizing wafer-level integration, packaging reduction, and improved energy utilization in complex system-on-chip platforms [31]. These developments reinforce the need to evaluate silicon anodes not only in terms of half-cell capacity, but also in terms of thermal budget, process contamination, alignment tolerance, encapsulation, and electrical integration with the target microsystem [1,2,5,6,7,8,9,30,31,32,33].

## 3. Planar Thin-Film Silicon Anodes

### 3.1. Physical Vapor and Chemical Vapor Deposition of Amorphous Silicon Films

Planar amorphous silicon films remain important because they are the simplest platform for studying stress evolution, artificial interphases, and solid-state stacking. Their process compatibility is high: sputtering and PECVD can define thickness, composition, and deposition temperature, while lithography can isolate the active area. The main limitation is that film thickness simultaneously affects capacity and mechanical risk. Very thin films can cycle reversibly because their strain energy is low; however, their areal capacity is often insufficient for autonomous microsystems. Thicker films increase areal capacity but accumulate larger stresses, making crack spacing, adhesion, current-collector roughness, and film microstructure decisive parameters [8,9,16,46,47,48,49,50,68,88,89,90].

The planar film is also a useful baseline for the evaluation of 3D structures. If a microstructured electrode offers only a modest gain in areal capacity but requires a much more complex fabrication sequence, planar films might remain preferable for low-power devices. Conversely, if the application requires higher energy per footprint, planar films show why 3D architectures are needed; adding thickness alone eventually degrades transport and reliability. Therefore, planar silicon should not be viewed as obsolete; it is a reference architecture that clarifies the trade-off between process simplicity, areal loading, stress, and integration with solid electrolytes [3,8,9,10,11,17,18,19,20,21,22,46,47,48,49,50].

The simplest microfabricated silicon anode is a planar amorphous silicon film deposited on a current collector [8,9,46,47,48,49,50]. Sputtering, evaporation, plasma-enhanced chemical vapor deposition, and low-pressure chemical vapor deposition can produce silicon films with controlled thickness, composition, and wafer-scale uniformity [3,8,9,17,18,19,20,21]. Thin-film silicon can be patterned by lift-off, etching, or shadow masking, allowing for direct definition of microbattery footprints and straightforward integration with thin-film electrolytes and cathodes [3,4,8,9,12,13,14,15,16,17,18,19,20,21].

Thin-film silicon anodes provide excellent process simplicity and compatibility with stacked solid-state microbattery concepts; however, they are limited by the trade-off between areal capacity and mechanical stability [8,9,46,47,48,49,50]. Films that are thin enough to avoid severe cracking often provide only a modest areal capacity, whereas thicker films generate larger stresses and are more susceptible to delamination [46,47,48,49,50]. Therefore, planar silicon films are particularly attractive for low-power thin-film microbatteries, whereas higher-energy applications often require patterned or three-dimensional architectures [5,6,7,8,9,32,33].

### 3.2. Patterned Silicon Islands, Stripes, and Microdisks

Patterned silicon islands provide a mechanically intuitive compromise between continuous films and fully three-dimensional architectures. By dividing the film into discrete domains, the design interrupts the long crack paths and allows each island to deform more independently. The spacing between islands can serve as expansion volume and an electrolyte-access channel, whereas the island dimensions control stress accumulation. However, the same spacing reduces active-material density; therefore, the pattern must be chosen by balancing mechanical durability against footprint-normalized capacity. In this sense, patterned islands are a lithographic solution to a mechanical problem, and their performance depends on both electrode chemistry and mask geometry [46,47,48,49,50,69,70,71].

In Figure 1b, the two current-collector patterning strategies highlight why patterned electrodes are attractive for on-chip cells. The lift-off route is convenient when a metal film is deposited after photoresist patterning, whereas the metal-etch route is better suited for cases where a blanket current collector is deposited first and selectively removed. Both approaches can define interdigitated layouts, isolated current collectors, or addressable test structures. For silicon anodes, such patternability enables systematic studies of island width, pitch, and contact geometry, which are difficult to achieve in slurry electrodes. The same lithographic precision can also be used to align anode and cathode features in a full microbattery stack [1,5,6,7,23,24,25,32,33].

Photolithographically patterned silicon islands, stripes, and microdisks can improve mechanical reliability by limiting crack propagation and reducing the lateral length scale over which stress accumulates [47,48,49,50]. In a continuous film, one crack can extend across the electrode, whereas in a patterned electrode, each island deforms more independently and failure is spatially localized [47,48,49,50]. The spacing between islands provides expansion volume and electrolyte access, but excessive spacing reduces active-material packing density and, therefore, footprint-normalized capacity [1,2,5,6,7,8,9,30,32,33].

Figure 1b further emphasizes that the lift-off and metal-etching routes provide complementary methods for defining microbattery electrode footprints with precise lateral geometries.

Patterned thin-film electrodes are an intermediate design between planar films and high-aspect-ratio 3D electrodes [1,2,5,6,7,8,9,32,33]. They are easier to fabricate and coat than deep microstructures, but they offer less areal-capacity enhancement than micropillars or trenches [5,6,7,8,9,32,33]. Therefore, their value is greatest when robust cycling, lithographic alignment, and process simplicity are more important than maximum areal loading [8,9,47,48,49,50].

## 4. Three-Dimensional Microstructured Silicon Anodes

### 4.1. Rationale for 3D Silicon Architectures

The central rationale behind using 3D silicon is that it decouples the footprint from the active area. A vertical pillar or trench array can provide multiple times the planar surface area within the same projected footprint, enabling higher areal capacity while retaining short local diffusion distances. However, the increased area also increases the number of interfaces that must remain stable. In liquid-electrolyte half cells, this may appear as a large irreversible capacity and SEI growth. In solid-state microbatteries, this appears as challenges related to conformality and interfacial resistance. Therefore, 3D silicon architectures are the most compelling when the additional surface area is paired with effective interface protection and a balanced full-cell design [2,3,4,5,6,7,8,9,10,11,12,13,14,15,16,17,18,19,20,21,22,30,32,33].

**Figure 2:**
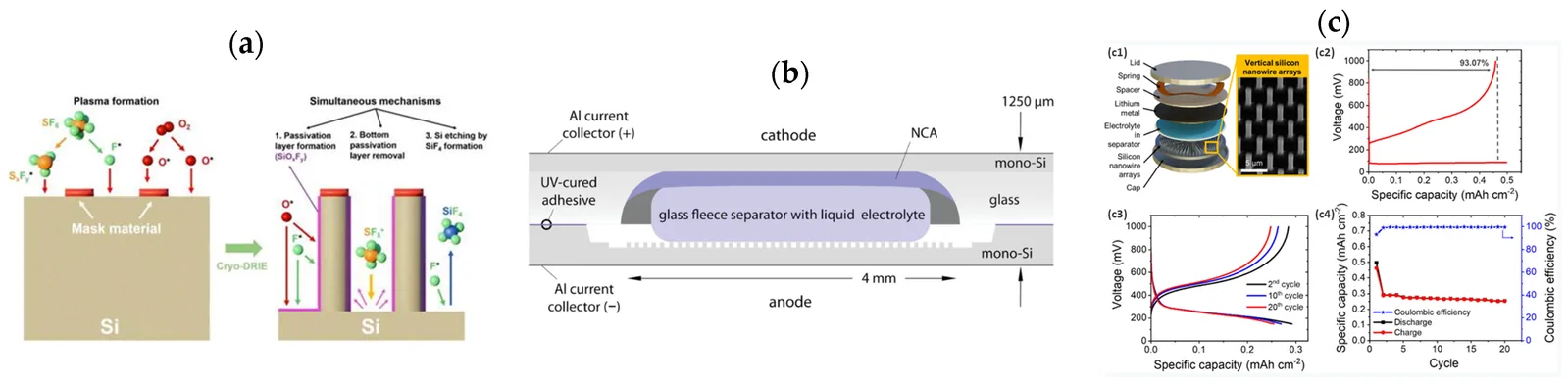
Fabrication, full-cell architecture, and electrochemical aspects relevant to three-dimensional Si-based microbatteries. (**a**) Cryogenic DRIE mechanism for anisotropic fabrication of high-aspect-ratio Si nanostructures [56]. (**b**) Cross-sectional architecture of a rechargeable lithium-silicon microbattery in which semiconductor-grade monocrystalline Si serves as the anode and structural housing [85]. (**c**) Li-ion half-cell configuration and electrochemical response of a vertically aligned Si nanowire-array anode [4]. Reproduced/adapted from Refs. [4,56,85], under the terms of the Creative Commons Attribution 4.0 International License. In panel (**a**), the colored species distinguish fluorine- and oxygen-containing reactants/intermediates, asterisks denote reactive radical species, and arrows indicate the passivation and etching pathways. For unambiguous reference, the four source-internal panels within panel (**c**) are designated (**c1**)–(**c4**), corresponding to the assembled half-cell/Si-nanowire array, the first-cycle voltage–capacity curve, selected-cycle voltage–capacity profiles, and cycling/Coulombic-efficiency behavior, respectively.

Figure 2a illustrates the cryogenic DRIE mechanism used to form anisotropic, high-aspect-ratio silicon nanostructures, highlighting a fabrication route directly relevant to three-dimensional silicon anodes [56]. The ability to control sidewall profile and feature geometry is important because these parameters influence active surface area, mechanical stability, and the conformality of subsequently deposited battery layers.

Three-dimensional silicon anodes are central to the development of high-energy on-chip microbatteries because they increase active-material loading and electrochemically accessible area without increasing the lateral footprint [2,5,6,7,8,9,30,32,33]. Recent reviews of 3D silicon-based lithium-ion microbatteries emphasize that device architecture, material compatibility, cell design, fabrication method, and application target are inseparable in determining microbattery performance [2,30]. A successful 3D silicon anode must provide a high surface area, continuous electron conduction, accessible ion transport, and sufficient free volume for expansion [2,5,6,7,8,9,30,32,33].

Figure 2a illustrates cryogenic DRIE as a route to high-aspect-ratio Si nanostructures, whereas Figure 2b shows a rechargeable lithium-silicon microbattery in which semiconductor-grade monocrystalline Si functions as both the anode and structural housing. Figure 2c then connects a vertically aligned Si nanowire array architecture with its electrochemical half-cell response. Together, these examples link microfabrication, device-level integration, and electrochemical validation in Si-based microbatteries [4,56,85].

The main difficulty with 3D architectures is their integration complexity [5,6,7,8,9,32,33]. High-aspect-ratio silicon structures can be fabricated using MEMS processes, but the electrolyte and cathode must be subsequently deposited or infiltrated into the same complex geometry [3,4,12,13,14,15,16,17,18,19,20,21]. Therefore, the most useful silicon architecture is not always the one with the highest theoretical area enhancement; rather, it is the one that can be uniformly coated, balanced with a cathode, sealed, and reliably operated in a full cell [1,2,5,6,7,8,9,30,31,32,33].

### 4.2. Deep Reactive Ion Etching

DRIE is particularly attractive because it uses tools already familiar in MEMS fabrication. Bosch DRIE alternates between fluorine-based etching and polymer passivation to create deep features with high anisotropy, while cryogenic DRIE uses low-temperature sidewall passivation to produce smooth profiles. These differences are not merely cosmetic for the Si anodes. Scalloped sidewalls can increase surface area but also create local stress concentrations and nonuniform coating thicknesses. Smooth cryogenic sidewalls may improve mechanical reliability and conformal coating, but the process window can be narrower and more sensitive to temperature, gas composition, and mask material [4,12,13,14,15,16,56,91,92,93].

**Figure 3:**
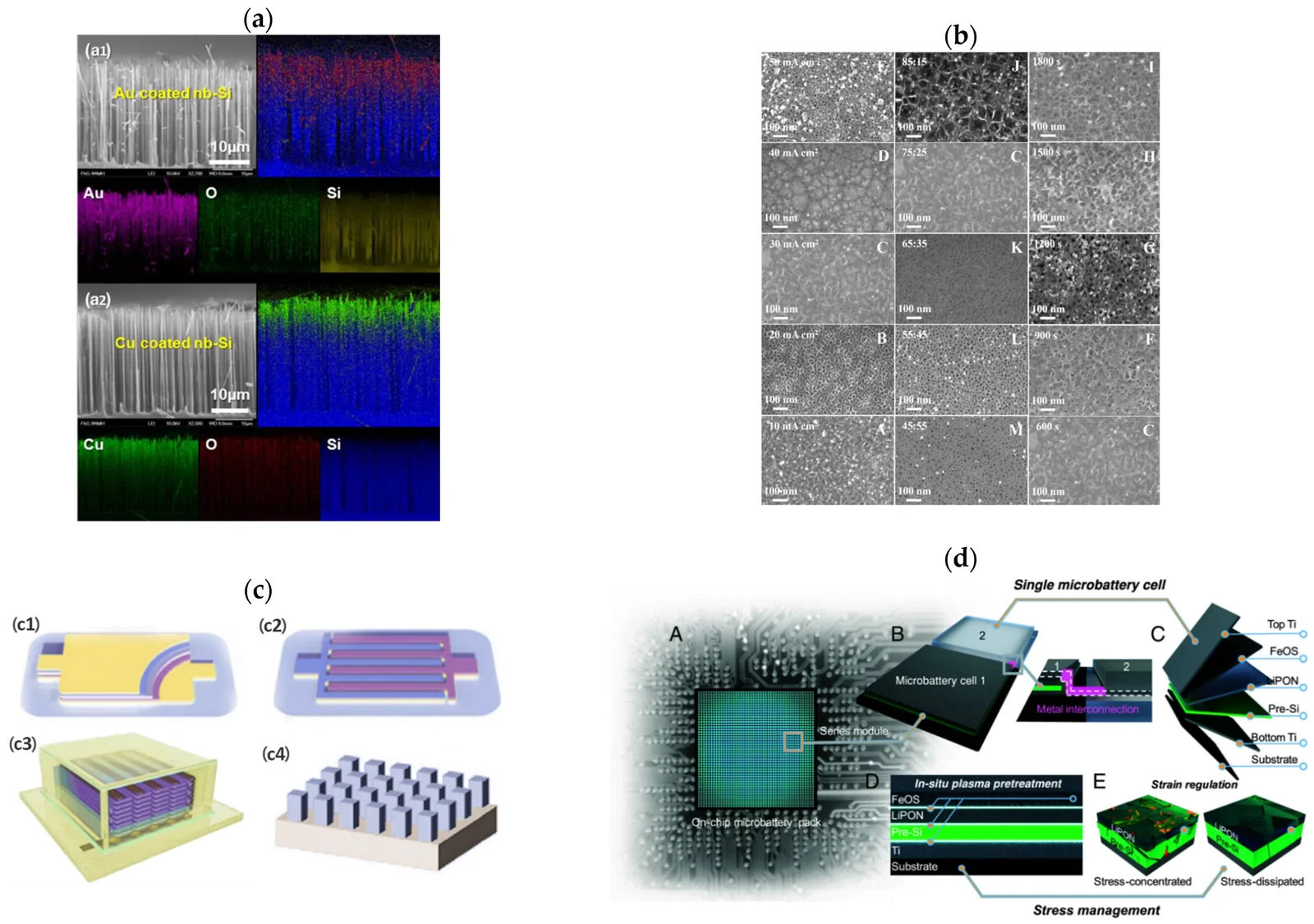
Process and architecture examples for microfabricated Si-anode and on-chip microbattery systems. (**a**) Cross-sectional morphology and elemental mapping of Cu-coated nanostructured black Si fabricated by ICP etching and sputter deposition for a CMOS-compatible Si microbattery anode [57]. (**b**) SEM images of self-standing mesoporous Si films fabricated under different electrochemical etching conditions, illustrating process-dependent control of porous-Si morphology [94]. (**c**) Schematic comparison of laminated thin-film, planar interdigital, three-dimensional interdigital, and microtube-type micro-LIB architectures [3]. (**d**) Schematic illustrations of an integratable all-solid-state thin-film microbattery system, including on-chip battery-pack, series-connected-cell, and single-cell configurations [81]. Panels (**a**–**c**) are reproduced from Refs. [3,57,94], respectively, under their applicable Creative Commons licenses, and panel (**d**) is reproduced without modification from Ref. [81] under the Creative Commons Attribution-NonCommercial-NoDerivatives 4.0 International License. Within panel (**a**), the two source-internal panels are designated (**a1**) and (**a2**) and correspond to Au-coated and Cu-coated nanostructured black Si, respectively. In panel (**b**), the letter annotations in the source micrographs are original sample identifiers associated with the displayed current-density, composition-ratio, and etching-time conditions rather than subpanel labels. The four source-internal schematics in panel (**c**) are designated (**c1**)–(**c4**) and correspond to laminated thin-film, planar interdigital, three-dimensional interdigital, and microtube-type architectures, respectively. In panel (**d**), labels A–E are source-schematic callouts for the wafer/battery-pack overview, single-cell module, layer stack, in-situ plasma pretreatment/stress-management concept, and strain-regulation concept. In the elemental maps, colors identify the labeled elements; schematic colors follow the material labels shown in the source artwork.

Figure 3 broadens the process-level discussion by illustrating how different microfabrication strategies translate into silicon morphology and, ultimately, into integrable microbattery architectures. Figure 3a shows an ICP-etched nanostructured black-Si anode with a conformal Cu coating, demonstrating the combination of surface-area enhancement and current-collector integration in a CMOS-compatible process [57]. Figure 3b further illustrates how electrochemical etching conditions can tune the morphology of self-standing mesoporous Si films [94]. Figure 3c and Figure 3d extend this process–structure relationship to the device level by comparing representative micro-LIB architectures [3] and an integratable all-solid-state thin-film microbattery concept [81], respectively. Together, these examples emphasize that silicon-anode microfabrication must be optimized not only for feature geometry but also for electrode integration, electrolyte/cathode compatibility, and the final on-chip cell architecture.

DRIE also raises practical sequencing questions for microbattery integration. If the silicon nanostructure is etched before current-collector metallization, metal coverage at the bottom and sidewalls may be nonuniform. If metallization is performed first, the metal must survive subsequent etch chemistry and thermal steps. If the architecture is intended for an all-solid-state cell, the DRIE profile must be compatible with electrolyte and cathode deposition. These constraints imply that an optimized etch recipe for a standalone silicon nanowire half-cell may not be directly transferable to a packaged on-chip microbattery. Therefore, process integration, including mask durability, etch rate, sidewall angle, loading effect, and post-etch surface cleaning, should be reported as part of the electrode design [3,4,10,11,12,13,14,15,16,17,18,19,20,21,22,91,92,93,95].

Deep reactive-ion etching is one of the most powerful tools for fabricating high-aspect-ratio silicon microstructures [4,12,13,14,15,16]. In the Bosch process of DRIE, alternating etching and passivation steps create deep trenches, holes, or pillars, whereas cryogenic DRIE can generate smoother sidewalls and finely controlled silicon nanowire morphologies [4,12,13,14,15,16]. These methods are already widely used in MEMS and through-silicon-via processing, making them attractive for wafer-scale fabrication of microbattery anodes [1,2,5,6,7,8,9,30,31,32,33].

For silicon anodes, DRIE enables the fabrication of micropillar arrays, microtrench arrays, and high-surface-area scaffolds with deterministic dimensions [4,12,13,14,15,16]. Pillar diameter, pitch, height, taper angle, and sidewall roughness affect active material loading, stress relaxation, electrolyte access, and coating conformality [4,5,6,7,8,9,12,13,14,15,16]. Cryogenic plasma processes have also been used to produce vertically aligned silicon nanowire arrays with controlled diameter and height, supporting the idea that dry etching can generate both microscale and nanoscale anode structures within a wafer-compatible process window [4,13,14,15,16].

**Figure 4:**
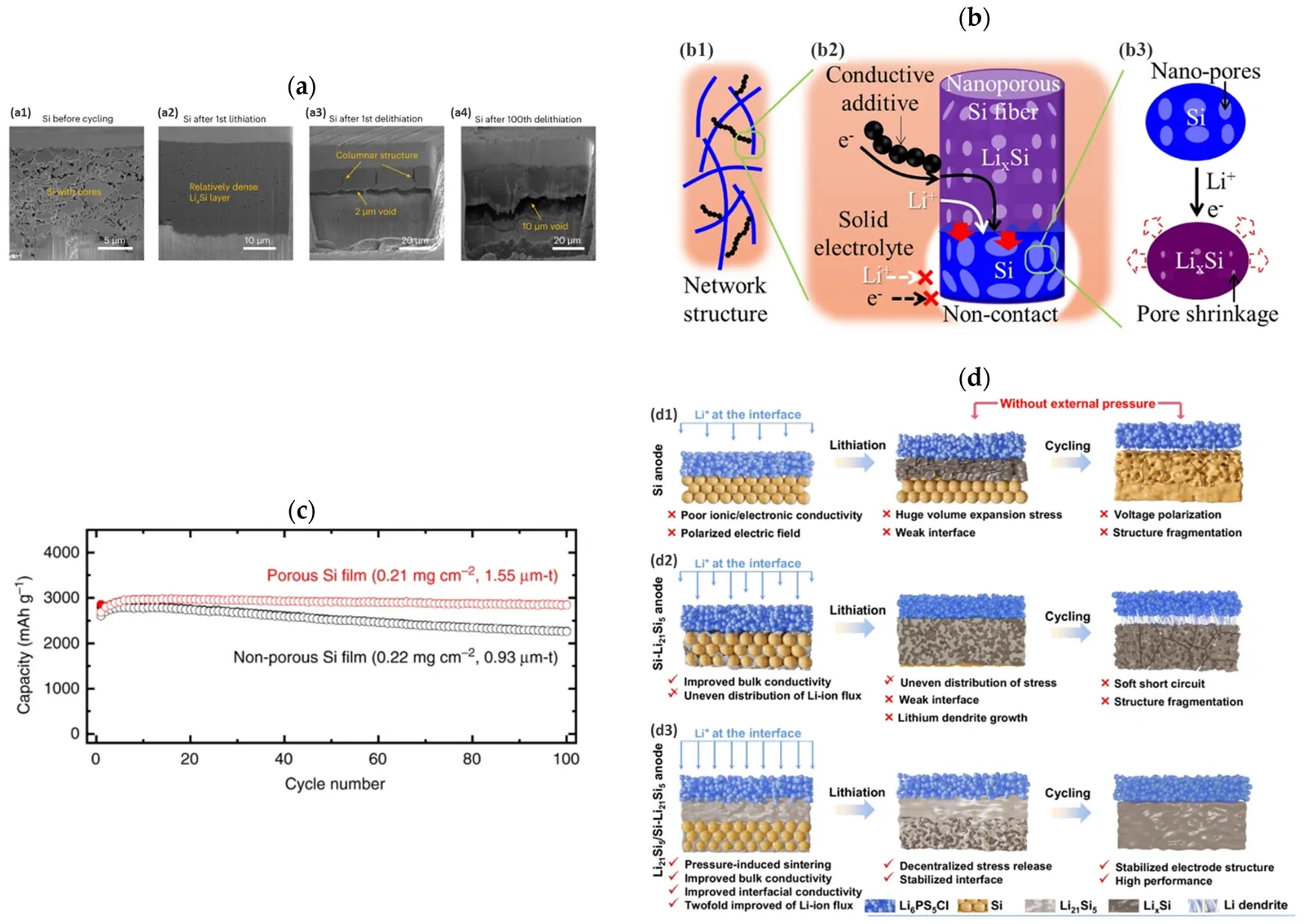
Mechanical degradation, structural accommodation, electrochemical stability, and advanced solid-state integration of Si anodes are shown. (**a**) Cross-sectional SEM images showing the evolution of a solid-electrolyte-free Si anode before cycling, after the first lithiation and delithiation, and after prolonged cycling, illustrating cycling-induced void formation and interfacial degradation [96]. (**b**) Schematic illustration of a composite anode based on a nanoporous Si-fiber network, highlighting continuous ionic/electronic pathways and accommodation of Si volume expansion through pore shrinkage [97]. (**c**) Cycling performance of non-porous and porous amorphous Si-film anodes, demonstrating enhanced cycling stability through porous-structure engineering [98]. (**d**) Schematic comparison of Si-based all-solid-state anode architectures, including a Li_21_Si_5_/Si–Li_21_Si_5_ double-layer structure designed to homogenize ionic/electronic transport and expansion stress for pressure-free operation [99]. Panels (**a**–**d**) are adapted from Refs. [96,97,98,99], respectively, under the terms of the Creative Commons Attribution 4.0 International License. For unambiguous reference, the four source-internal images in panel (**a**) are designated (**a1**)–(**a4**), corresponding to Si before cycling, after the first lithiation, after the first delithiation, and after the 100th delithiation, respectively. The three source-internal schematics in panel (**b**) are designated (**b1**)–(**b3**) and illustrate the network structure, local transport/non-contact region, and pore-shrinkage mechanism. The three source-internal rows in panel (**d**) are designated (**d1**)–(**d3**) and compare the Si anode, Si–Li21Si5 anode, and Li21Si5/Si–Li21Si5 architecture, respectively. Colored curves, symbols, and structural elements distinguish the compared data series and architectures as labeled in the source panels.

Figure 4 connects the mechanical degradation of Si anodes with structural strategies for improving electrochemical and interfacial stability. The progressive formation of interfacial voids during repeated lithiation and delithiation illustrates the severe chemo-mechanical degradation that can occur at Si/solid-electrolyte interfaces (Figure 4a) [96]. Nanoporous Si-fiber networks provide a strategy for mitigating this problem by accommodating volume expansion through pore shrinkage while maintaining continuous ionic and electronic pathways (Figure 4b) [97]. Consistent with this concept, porous amorphous Si films exhibit substantially improved cycling stability compared with non-porous films (Figure 4c) [98]. At the device level, advanced Si-anode architectures can further control ionic/electronic transport and stress distribution, as demonstrated by the Li_21_Si_5_/Si–Li_21_Si_5_ double-layer design for pressure-free all-solid-state batteries (Figure 4d) [99]. These results emphasize that reliable Si-based microbatteries require coordinated control of volume accommodation, interfacial contact, charge-transport pathways, and cell-level architecture.

### 4.3. Silicon Micropillars, Microtrenches, and Hollow Structures

Micropillars and microtrenches represent two different strategies for exploiting vertical dimensions. Pillars provide isolated expansion units with open sidewalls, which can reduce mechanical coupling between neighboring features if the pitch is sufficient. Trenches provide continuous vertical surfaces that may be easier to connect electrically and align lithographically; however, their narrow channels can be difficult to fill with solid electrolyte or cathode material. Hollow or tubular structures go one step further by adding an internal void volume, which allows for inward and outward expansion. These structures can improve mechanical accommodation but require more complex fabrication and often introduce additional interfaces that must remain stable during cycling [2,4,5,6,7,8,9,12,13,14,15,16,30,51,52,53,54,55].

The key design variables for these 3D structures are diameter or trench width, pitch, height, taper, sidewall roughness, and top-surface coverage. Large features are mechanically robust and easier to coat but provide lower area enhancement, while small features increase area and reduce diffusion length but may collapse, agglomerate, or become difficult to wet. In a full microbattery, the cathode must be engineered around the anode geometry. A high-aspect-ratio anode is not useful if the cathode cannot provide a matching capacity or if the electrolyte layer becomes discontinuous. Therefore, micropillar and trench designs should be evaluated in terms of full-stack compatibility, particularly the ability to achieve conformal electrolyte and cathode integration over the three-dimensional geometry, rather than as isolated anode structures [3,7,18,76,77].

Silicon micropillars are considered intuitive 3D anode designs because each pillar is electrically connected to the substrate, while lithium ions access the sidewalls [2,4,5,6,7,8,9,12,13,14,15,16,30]. The void space between the pillars accommodates lithiation expansion and facilitates electrolyte penetration [4,5,6,7,8,9,12,13,14,15,16]. However, dense or tall pillars can become difficult to wet and coat uniformly, and excessively narrow spacing can lead to mechanical interaction between neighboring structures during swelling [4,5,6,7,8,9,12,13,14,15,16,51,52,53,54,55].

Microtrenches and microchannels exploit the vertical sidewall area within the silicon substrate [2,4,5,6,7,8,9,12,13,14,15,16,30]. Compared with isolated pillars, trenches can provide continuous surfaces that are easier to align with planar current collectors and stacked layers; however, they may be difficult to fill uniformly with solid electrolyte or cathode material [3,4,12,13,14,15,16,17,18,19,20,21]. Hollow silicon structures and silicon microtubes add an internal void volume, allowing inward and outward expansion; however, they typically require more complex sacrificial templates or release processes [5,6,7,8,9,39,51,52,53,54,55].

### 4.4. Interdigitated and Monolithic 3D Cell Layouts

Interdigitated layouts are particularly relevant for microscale batteries because they replace long through-thickness ion transport with shorter lateral transport between neighboring electrode fingers. This geometry can deliver high power when the electrode spacing is small, and the electrolyte conductivity is sufficient. For silicon anodes, an interdigitated architecture allows lithographic registration between the silicon-containing anode and cathode fingers. However, close spacing increases the risk of shorts, nonuniform current distribution, and edge effects. Therefore, the electrolyte or separator must be precisely placed, and the electrode heights must be balanced to avoid underutilized materials [5,6,7,32,33,100,101,102].

A monolithic layout may ultimately be more practical than assembling separate microbattery components because it reduces packaging volume and alignment error. In this scheme, the silicon wafer can serve as the substrate, mechanical support, and active anode, whereas the current collectors, electrolyte, cathode, and encapsulation are sequentially deposited. The difficulty is that each step must be compatible with the materials already present. For example, the cathode crystallization temperature may damage polymeric layers, electrolyte deposition may not cover high-aspect-ratio features uniformly, and encapsulation must seal complex topography. These stack-level constraints should be considered part of the anode design strategy [1,2,3,7,8,9,30,31,80,81,82,85,86,87].

Interdigitated microbattery layouts place anode and cathode fingers laterally side by side on the same substrate, reducing the ion-transport distance and allowing for relatively thick electrodes within a small footprint [5,6,7,32,33]. High-performance interdigitated lithium-ion microbatteries have been demonstrated using 3D printing, holographic patterning, and bicontinuous electrode architectures, illustrating the importance of geometry in microbattery performance [5,6,7,32,33]. Although not all of these reports involve silicon-anode systems, their design principles are highly relevant to silicon because the anode can be patterned directly from the silicon substrate or deposited as a silicon-containing microelectrode [5,6,7,8,9,32,33].

The main integration challenge in interdigitated silicon microbatteries is maintaining electrical isolation, uniform electrolyte coverage, cathode/anode capacity balance, and hermetic packaging in a complex lateral geometry [5,6,7,8,9,32,33]. Edge effects and nonuniform current distribution can become significant when electrode fingers are closely spaced; therefore, electrochemical modeling and layout-level design are important for optimizing power density and cycle life [5,6,7,8,9,32,33,100,101,102].

## 5. Silicon Nanowire, Nanopillar, Porous-Silicon, and Black-Silicon Anodes

### 5.1. One-Dimensional Silicon Nanostructures

One-dimensional silicon nanostructures are among the most widely studied silicon-anode geometries because their small diameters enable radial strain relaxation. The seminal silicon-nanowire concept demonstrated that a direct electrical connection to the substrate and free radial expansion can mitigate pulverization compared with bulk particles. The same concept is attractive for microbatteries because nanowires can be grown or etched directly on wafers. However, a large surface area produces more SEI, and very long nanowires can bend, bundle, or lose contact with one another. Therefore, nanowire length and pitch should be selected based on an areal energy target rather than simply being maximized [4,10,11,13,14,15,16,23,24,25,35,36,37,38,39,40,41,42,43,44,45,100,101,102].

Figure 2c links nanowire architecture to electrochemical behavior by showing a half-cell with vertical silicon nanowire arrays and the corresponding charge–discharge response. This connection is important because morphology alone does not prove microbattery utility. A nanowire array must deliver stable capacity per footprint, maintain Coulombic efficiency, and preserve mechanical integrity after cycling. When the first-cycle irreversible capacity is high, the full-cell penalty may be severe because the cathode has limited lithium inventory. Thus, nanowire electrodes should be reported with both half-cell metrics and full-cell implications, including the need for prelithiation, the SEI stabilization strategy, and the cathode-capacity balance requirement [4,26,27,41,42,43,44,45,62,63,64].

Silicon nanowires and nanopillars have been widely studied owing to their small lateral dimensions, which provide free surfaces for strain relaxation during lithiation [35,36,37,38,39,40,41,42,43,44,45]. Vertically aligned silicon nanowire arrays can maintain direct electrical connection to the substrate while allowing radial expansion, which reduces the probability of catastrophic pulverization compared with bulk silicon particles [35,36,37,38,39,42,43,44,45]. This architecture is particularly attractive for microbatteries because nanowires can be fabricated on wafers and integrated with planar current collectors or solid-electrolyte coatings [4,5,6,7,8,9,12,13,14,15,16].

In Figure 2c, the half-cell test of a vertical silicon nanowire-array anode connects the microfabricated electrode architecture to electrochemical outputs, such as discharge–charge behavior, capacity retention, and Coulombic efficiency.

The electrochemical performance of nanowire arrays depends on their diameter, length, pitch, crystallinity, doping, surface chemistry, and coating strategy [4,13,14,15,16,35,36,37,38,39,40,41,42,43,44,45]. Smaller diameters reduce diffusion length and mechanical stress but increase surface area; therefore, irreversible lithium consumption associated with SEI formation increases [41,42,43,44,45,56,57,58,59,60,61]. Longer nanowires increase areal capacity but may bend, bundle, collapse, or become difficult to coat uniformly [4,5,6,7,8,9,12,13,14,15,16]. Consequently, nanowire design requires coordinated optimization of capacity, surface reactivity, mechanical stability, and manufacturability [2,4,5,6,7,8,9,12,13,14,15,16,30].

### 5.2. Metal-Assisted Chemical Etching (MACE) and Porous Silicon

MACE and related porous-silicon routes offer a low-cost path to high-area silicon because they can create vertical pores, nanowires, or black silicon surfaces without the same equipment burden as advanced lithography. This method is attractive for wafer-scale production because etch depth and morphology can be tuned through catalyst deposition, oxidant concentration, HF chemistry, doping, and etch time. From a battery perspective, porous silicon provides internal void space that can buffer expansion and shorten ion paths. The drawback is that the random structure may be mechanically fragile, and residual noble-metal catalysts must be removed or controlled to avoid contamination and parasitic reactions [13,14,15,16,65,66,67,103,104,105].

Porous silicon also illustrates a trade-off between surface area and interface stability. A large internal area improves electrolyte access and can increase utilization; however, it also increases the amount of SEI formed during the first cycle. In a half cell, this loss may be masked by lithium metal, whereas in a full microbattery, it directly consumes cathode lithium and lowers energy density. Protective carbon, oxide, polymer, or ALD coatings can reduce the penalty, but the coating must penetrate the pores uniformly without blocking transport. Therefore, pore size distribution, open porosity, tortuosity, and coating conformality should be reported together with electrochemical performance [3,17,18,19,20,21,41,42,43,44,45,56,57,58,59,60,61,94,103,104,105].

Metal-assisted chemical etching is a versatile wet-chemical method for fabricating silicon nanowires, pores, and high-surface-area silicon scaffolds [13,14,15,16]. In typical MACE processes, noble-metal catalysts locally promote silicon oxidation and dissolution in HF-containing solutions, leaving behind vertical pores or nanowire arrays [13,14,15,16]. This method can produce high-aspect-ratio structures over large areas using relatively simple equipment and at a lower cost than advanced lithography [13,14,15,16].

From a manufacturing standpoint, MACE should be treated as a contamination-controlled wet process rather than simply as a low-cost substitute for DRIE. Residual Ag or Au catalyst may pose contamination concerns in a shared semiconductor process line, and incomplete catalyst removal can also alter interfacial chemistry. Etch depth and porosity may vary with catalyst coverage, substrate doping, HF/H_2_O_2_ transport, local bubble removal, and etch time, which creates across-wafer nonuniformity. Therefore, MACE may be better suited to a dedicated or post-CMOS module in which catalyst removal is verified and etch-depth/porosity statistics are reported across the wafer [13,14,15,16,103,104,105].

### 5.3. Lithographically Defined Nanopillars and Nanoimprint Routes

Lithographically defined nanopillars provide a bridge between deterministic MEMS processing and nanoscale strain engineering. Compared with randomly etched porous structures, lithographic patterns allow researchers to vary diameter, pitch, and array symmetry independently, making it easier to identify the geometry that minimizes fracture or maximizes rate capability. Nanoimprint lithography and colloidal masks may be especially useful when large-area periodic patterns are required at a lower cost than electron-beam lithography. The limitation is that each additional patterning step increases process complexity and may introduce residue, mask erosion, or alignment constraints [4,12,13,14,15,16,23,24,25,91,92,93,95].

In practical manufacturing, the most valuable role of lithographically defined nanostructures is parametric learning rather than direct mass production. Well-controlled arrays can reveal how silicon diameter affects fracture, how pitch affects electrolyte access, and how sidewall morphology affects the coating. The design rules obtained from these studies can then guide more scalable processes, such as nanoimprint lithography, MACE, or plasma texturing. Thus, deterministic nanopillar studies can serve as a design platform for translating fundamental silicon mechanics into manufacturable microbattery architectures [43,44,45,46,47,48,49,50,100,101,102].

Lithographically defined nanopillars provide more precise geometry control than randomly etched porous structures [1,2,4,12,13,14,15,16]. Electron-beam lithography, nanoimprint lithography, nanosphere lithography, colloidal masks, and advanced photolithography can define submicrometer patterns, which are transferred into silicon by reactive ion etching or deep reactive ion etching [4,12,13,14,15,16]. This approach enables systematic studies on how the diameter, pitch, sidewall morphology, and aspect ratio influence silicon-anode failure mechanisms [4,5,6,7,8,9,12,13,14,15,16,46,47,48,49,50,51,52,53,54,55].

For practical manufacturing, photolithography and nanoimprint lithography are more scalable than electron-beam lithography [1,4,12,13,14,15,16]. Nanosphere and colloidal masks offer low-cost periodic patterning but provide less design freedom [13,14,15,16]. The choice of patterning method should be guided by the target device area, acceptable cost, alignment tolerance, and compatibility with the rest of the microbattery stack [1,2,5,6,7,8,9,30,31,32,33].

## 6. Interface, Coating, and Current-Collector Strategies

### 6.1. Carbon Coatings and Conductive Networks

Carbon coatings are valuable because they combine electronic conductivity with partial chemical protection. In nanowire and porous-silicon electrodes, a conformal carbon shell can maintain electrical continuity during expansion and reduce the direct exposure of silicon to the electrolyte. However, the carbon layer must be engineered carefully: if it is too thin, it may crack or fail to suppress SEI growth; if it is too thick, it adds inactive volume and increases lithium-ion transport resistance. In microbatteries, this inactive-volume penalty is particularly important because the packaged device volume is small [40,41,42,43,44,45,52,53,54,55,56,57,58,59,60,61,106,107,108].

The conductive network also changes the failure modes. Instead of losing capacity through pulverization alone, a carbon-coated silicon scaffold may fail through shell fracture, delamination between carbon and silicon, pore blocking by SEI, or loss of contact at the current collector. Consequently, the success of a coating strategy should be evaluated based on the post-cycling morphology and impedance, and not only on the initial capacity. Cross-sectional imaging is especially important for high-aspect-ratio electrodes because a coating that appears continuous from the top surface may be non-uniform deep inside trenches or nanowire forests [3,17,18,19,20,21,56,57,58,59,60,61,106,107,108].

Carbon coatings improve electrical conductivity, reduce direct electrolyte contact with silicon, and help stabilize the solid-electrolyte interphase [40,41,42,43,44,45,56,57,58,59,60,61]. In microfabricated silicon anodes, carbon can be deposited by chemical vapor deposition, pyrolysis of polymer precursors, sputtering, or solution processing [56,57,58,59,60,61]. Conformal carbon is particularly useful for nanowires and porous silicon because it helps maintain electronic connectivity during repeated expansions and contractions [40,41,42,43,44,45,56,57,58,59,60,61].

The carbon layer must be thin enough to avoid excessive inactive mass and ion transport resistance but robust enough to remain intact during cycling [56,57,58,59,60,61]. Uniform coating of high-aspect-ratio silicon is challenging, particularly within dense nanowire forests or deep trenches [4,12,13,14,15,16,56,57,58,59,60,61]. Therefore, carbon-coating strategies should be evaluated using both electrochemical performance and cross-sectional structural analysis after cycling [41,42,43,44,45,56,57,58,59,60,61].

### 6.2. Metals, Silicides, and Adhesion Layers

Current-collector design is a central issue for microfabricated silicon anodes because the current collector is not just a passive support. It defines contact resistance, adhesion, diffusion barriers, thermal stability, and process compatibility. Metals such as Cu, Ti, Pt, Ni, and Au exhibit high conductivity; however, their adhesion to silicon, reactivity with lithium, and compatibility with downstream processes differ. Silicides can provide stable, low-resistance contacts, whereas TiN and related barrier layers can limit diffusion. Therefore, the best stack depends on whether the electrode is a planar film, an etched silicon scaffold, or a fully integrated 3D cell [5,6,7,8,9,78,79,80,81,82,83,84].

In a small cell, contact loss can be as damaging as active-material fracture. A silicon pillar may remain structurally intact after cycling but may become partially disconnected if the metallization cracks, diffuses, or delaminates. Conversely, a robust current collector can maintain electronic connectivity in a damaged silicon network and improve usable capacity. Therefore, microbattery reports should include details of current-collector thickness, adhesion layer, annealing, diffusion barrier, and contact geometry. These details are often omitted in battery-focused studies but are essential for translating silicon anodes into wafer-level devices [1,2,5,6,7,8,9,30,31,78,79,80,81].

Metal current collectors and silicide contacts are important for microfabricated silicon anodes because they provide low-resistance electrical pathways and can improve adhesion [5,6,7,8,9,78,79,80,81]. Titanium, chromium, nickel, copper, platinum, and titanium nitride have been used as current collectors, adhesion layers, and diffusion barriers in silicon-based microbattery structures [5,6,7,8,9,78,79,80,81]. Metal-stabilized nanostructured silicon anodes have demonstrated the value of integrating battery chemistry with CMOS-oriented metallization concepts [78].

The metal layer must be selected based on its electrochemical stability, lithium-alloying behavior, diffusion, contamination, and thermal budget [5,6,7,8,9,78,79,80,81]. Copper offers high conductivity but may require diffusion barriers; titanium and titanium nitride provide adhesion and barrier functionality; and nickel silicide and other silicides can improve electrical contact with silicon [78,79,80,81]. Because the current collector occupies a significant fraction of the microbattery volume, its geometry and thickness should be included in device-level energy-density calculations [1,2,5,6,7,8,9,28,29,30,31,32,33].

### 6.3. Atomic Layer Deposition and Artificial Interphases

ALD is particularly suitable for microfabricated silicon because it can coat nonplanar and high-aspect-ratio structures with sub-nanometer thickness control [3,17,18,19,20,21,109,110]. Ultrathin Al2O3, TiO2, lithium-containing oxides, phosphates, or hybrid layers can act as artificial interphases that suppress continuous electrolyte decomposition and improve Coulombic efficiency. For high-aspect-ratio electrodes, the advantage of ALD is its conformality, while the challenges are throughput and precursor diffusion. Long exposure times may be required to uniformly coat deep trenches or dense nanowire arrays, and the resulting layer must remain mechanically intact during the silicon expansion [3,17,18,19,20,21,48,49,50,51,111,112,113]. These coatings can suppress continuous electrolyte decomposition, stabilize interfacial chemistry, and improve Coulombic efficiency and cycling stability [3,17,18,19,20,21,110,114].

The electrochemical role of an ALD layer can change during cycling. Some oxide layers are initially insulating but become lithiated and ionically conductive, whereas others remain resistive if they are too thick. Therefore, a successful artificial interphase must satisfy conflicting requirements: it must be sufficiently electronically blocking to reduce electrolyte decomposition, ionically permeable to allow lithium transport, mechanically compliant to survive expansion, and sufficiently thin to avoid a large inactive volume. For silicon-anode microbatteries, ALD coatings are most powerful when integrated into a complete stack that also includes a stable current collector and a compatible electrolyte [3,17,18,19,20,21,48,49,50,51,111,112,113].

Atomic layer deposition is especially valuable for microfabricated silicon anodes because it provides conformal nanoscale coatings on high-aspect-ratio structures [3,17,18,19,20,21]. ALD Al_2_O_3_, TiO_2_, lithium-containing oxides, phosphates, and other ultrathin layers have been explored as artificial interphases, mechanical coatings, and solid-electrolyte components [3,17,18,19,20,21]. These coatings can suppress continuous electrolyte decomposition, reduce irreversible lithium loss, and improve cycling stability [3,17,18,19,20,21].

**Figure 5:**
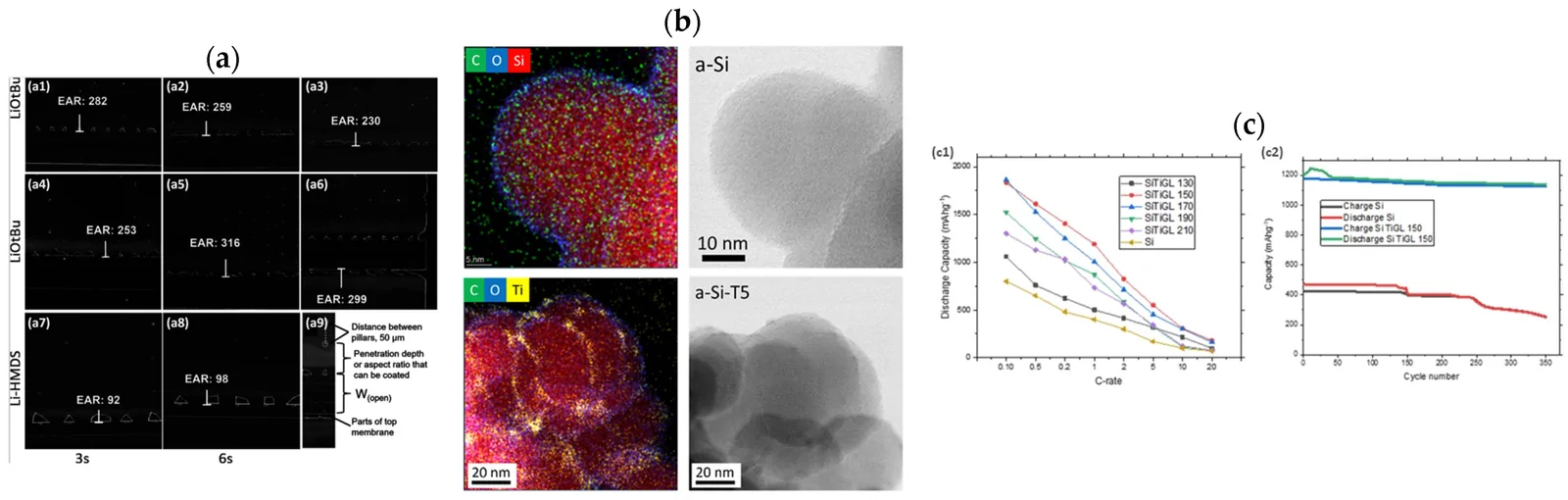
Conformal interface-engineering strategies for Si-based microbattery electrodes. (**a**) SEM-based evaluation of ALD-grown LiPON penetration into lateral high-aspect-ratio structures, demonstrating the influence of precursor and pulse conditions on coating conformality [109]. (**b**) TEM images and elemental maps comparing pristine amorphous Si nanoparticles with conformally TiO_2_-coated Si prepared by atomic layer deposition, demonstrating the formation of an artificial interphase on the Si surface [110]. (**c**) Rate capability and long-term cycling performance of uncoated and titanicone-coated Si electrodes, illustrating the improvement in electrochemical kinetics and cycling stability achieved through conformal ALD/MLD interface engineering [114]. Panels (**a**) and (**c**) are adapted from Refs. [109,114], respectively, under the Creative Commons Attribution 4.0 International License; panel (**b**) is adapted from Ref. [110] under the Creative Commons Attribution 3.0 Unported Licence. For unambiguous reference, the nine source images/schematic elements within panel (**a**) are designated (**a1**)–(**a9**) from left to right and top to bottom; the displayed source annotations specify the precursor/pulse conditions and extracted aspect-ratio values. The two source-internal plots within panel (**c**) are designated (**c1**) and (**c2**) and show rate capability and long-term cycling, respectively. In panel (**b**), elemental-map colors correspond to the labeled C, O, Si, and Ti channels; colored curves in panel (**c**) distinguish the compared electrodes as labeled in the source plots.

Figure 5 illustrates interface engineering from conformal thin-film deposition to its structural and electrochemical consequences. High-aspect-ratio test structures demonstrate that ALD precursor transport and pulse conditions directly determine the penetration depth and conformality of solid-electrolyte coatings (Figure 5a) [109]. At the Si surface, ALD can form nanometer-scale artificial interphases, as demonstrated by the conformal TiO_2_ coating surrounding amorphous Si nanoparticles in Figure 5b [110]. Such interface modification can translate into substantially improved electrochemical behavior: the titanicone-coated Si electrode in Figure 5c exhibits enhanced rate capability and markedly improved long-term capacity retention compared with uncoated Si [114]. These results emphasize that coating conformality, interfacial chemistry, and mechanical integrity must be optimized together when applying ALD- or MLD-based interface engineering to microfabricated Si anodes.

The thickness and chemistry of an ALD coating must be carefully optimized [3,17,18,19,20,21]. A film that is too thin may crack or fail to protect the silicon, whereas a film that is too thick may impede lithium transport and reduce accessible capacity [3,17,18,19,20,21]. Some oxide coatings become lithium-ion conductors after electrochemical activation, while others remain resistive; therefore, electrochemical impedance and post-cycling microscopy are essential for evaluating coating effectiveness [3,17,18,19,20,21].

### 6.4. Solid-State Electrolyte Integration

Solid-state electrolytes are attractive for use in on-chip batteries because they reduce leakage, improve packaging compatibility, and can be deposited as thin films. LiPON is the benchmark because of its stability and established history in thin-film batteries; however, integrating high-capacity silicon with rigid solid electrolytes remains difficult. Silicon expansion can fracture the electrolyte, create interfacial voids, or produce localized current constriction. These problems become more severe in 3D architectures where the electrolyte must coat curved or vertical surfaces uniformly [3,8,9,10,11,17,18,19,20,21,22,58,59,60,61].

Therefore, a practical solid-state silicon microbattery may require constrained lithiation rather than the full utilization of silicon’s theoretical capacity. By limiting the depth of discharge, using thin silicon domains, inserting compliant interlayers, or introducing engineered void space, the cell can trade some capacity for a longer lifetime and improve safety. This trade-off is reasonable for on-chip applications because reliability and packaging compatibility may be more valuable than the maximum half-cell capacity. Future work should report not only the initial capacity but also interfacial impedance growth, short-circuit statistics, and post-cycling electrolyte integrity [3,8,9,10,11,17,18,19,20,21,22,37,38,39,40,41,42,43,44,45,80,81,86,87].

All-solid-state microbatteries are attractive for on-chip applications because they reduce leakage risk, improve safety, and can be deposited as thin films [3,8,9,10,11,17,18,19,20,21,22]. LiPON remains a benchmark solid electrolyte for thin-film batteries because of its stability and long history in integrated microbattery research [8,9,10,11,22,115,116]. However, rigid solid electrolytes can fracture or debond if they are placed directly on silicon structures that undergo large lithiation-induced expansion [3,10,11,17,18,19,20,21,22,37,38,39,40,41,42,43,44,45].

For silicon-anode solid-state microbatteries, mechanical compatibility at the silicon/electrolyte interface is as important as ionic conductivity [3,10,11,17,18,19,20,21,22,37,38,39,40,41,42,43,44,45]. Possible design strategies include limiting the silicon thickness, using nanostructured silicon with controlled void space, adding compliant interlayers, forming artificial SEI coatings before solid-electrolyte deposition, and restricting the depth of lithiation during operation [3,10,11,17,18,19,20,21,22,37,38,39,40,41,42,43,44,45].

## 7. Printed, Laser-Patterned, and Hybrid Silicon Microelectrodes

Printed silicon electrodes provide a complementary route when low-cost patterning, flexible substrates, or rapid prototyping are more important than maximum volumetric energy density. Silicon nanoparticle inks can be deposited using inkjet printing, aerosol jet printing, screen printing, or dispenser printing, and the printed patterns can be aligned with lithographically defined current collectors. This hybrid approach combines the geometric precision of microfabrication with the material flexibility of composite electrodes. The penalty is that binders, carbon additives, and porosity reduce the volumetric energy density compared with directly etched silicon [5,6,7,23,24,25,26,32,33].

Laser processing can locally ablate, sinter, texture, or carbonize electrode materials without a mask. For silicon microelectrodes, laser patterning may be useful for creating local porosity, opening channels, or defining current-collector regions after deposition. However, the thermal effects must be carefully managed when the battery is integrated near electronics or polymer packaging. Hybrid approaches are likely to be the most practical; lithography can define high-resolution conductors and isolation regions, while printing or laser processing can add active material where high loading is needed [23,24,25,26].

Printing methods such as inkjet printing, aerosol jet printing, extrusion-based 3D printing, and stereolithography can pattern silicon-containing composite electrodes without conventional lithographic processing [5,6,7,23,24,25,32,33,117,118,119]. Silicon nanoparticles, conductive carbon or graphene, polymeric binders, and rheological modifiers can be formulated as inks, slurries, filaments, or photocurable resins, depending on the selected printing route [23,24,25,117,118,119]. These approaches are attractive for rapid prototyping, customized layouts, and flexible or nonplanar substrates [5,6,7,23,24,25,32,33].

**Figure 6:**
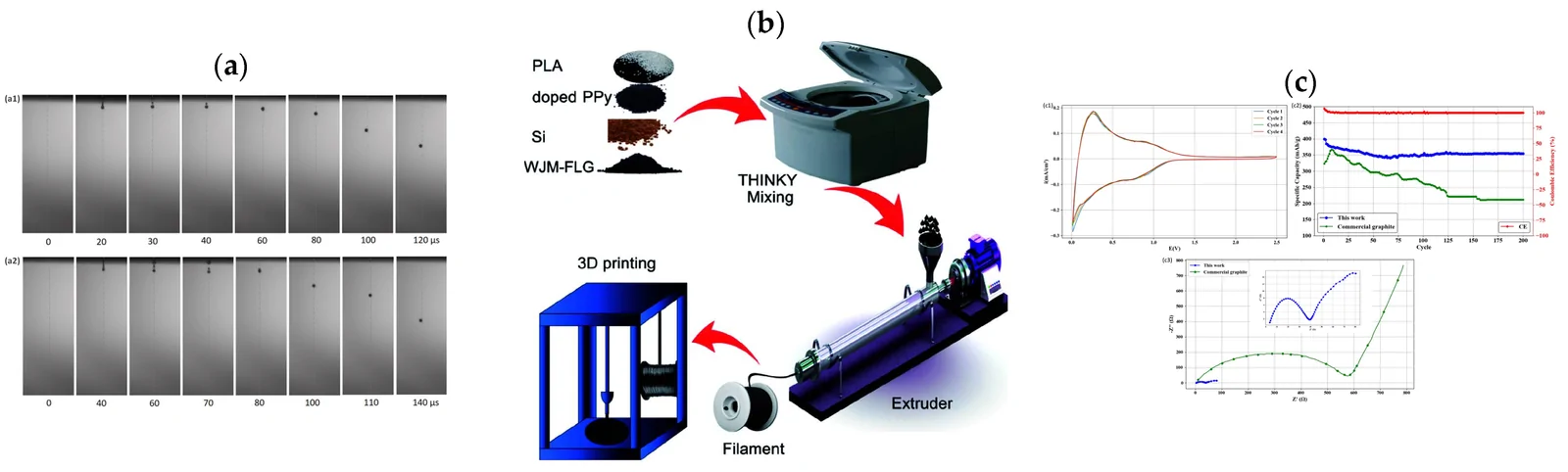
Additive-manufacturing routes and electrochemical validation of silicon-containing battery electrodes. (**a**) Frame-by-frame visualization of drop generation from Si-based anode inks during piezoelectric drop-on-demand inkjet printing, illustrating the role of ink formulation and jetting stability in printable-electrode fabrication [117]. (**b**) Schematic fabrication route for a Si/few-layer-graphene composite anode, including conductive-filament preparation and fused-deposition-modeling (FDM) 3D printing [118]. (**c**) Cyclic voltammetry, specific capacity, and impedance responses of a 3D-printed Si-containing anode prepared from recycled photovoltaic silicon, demonstrating electrochemical functionality after printing and pyrolysis [119]. Panel (**a**) is adapted from Ref. [117] under the Creative Commons Attribution 4.0 International License; panels (**b**) and (**c**) are adapted from Refs. [118,119], respectively, under the Creative Commons Attribution 3.0 Unported Licence. For unambiguous reference, the two source-internal drop-generation sequences within panel (**a**) are designated (**a1**) and (**a2**). The three source-internal plots within panel (**c**) are designated (**c1**)–(**c3**) and show cyclic voltammetry, capacity/cycling performance, and impedance response, respectively. These caption designations distinguish the source-internal labels from the top-level panels (**a**–**c**).

Figure 6 illustrates the progression from printability control to three-dimensional electrode fabrication and electrochemical validation. Drop-on-demand inkjet printing requires stable Si-containing inks and controlled droplet formation; deviations in droplet trajectory or nozzle instability can directly limit pattern fidelity and processing throughput (Figure 6a) [117]. Fused-deposition modeling provides a complementary route, in which Si nanoparticles, conductive additives, and a polymer matrix are first formulated into printable filaments and are subsequently shaped into three-dimensional electrodes (Figure 6b) [118]. More recently, stereolithography-based printing has also been applied to Si-containing anodes, including electrodes derived from recycled photovoltaic silicon, demonstrating that geometrically defined printed structures can retain useful electrochemical activity after post-printing thermal processing (Figure 6c) [119]. Together, these examples show that successful additive manufacturing of Si anodes requires coordinated optimization of feedstock formulation, printing stability, structural integrity, electrical connectivity, and electrochemical utilization.

Printed silicon electrodes often contain binder and conductive carbon; therefore, they may have lower volumetric energy density than directly etched silicon architectures [23,24,25,30]. Nevertheless, printing can complement microfabrication by filling trenches, depositing cathodes or gel electrolytes, or creating hybrid electrodes on pre-patterned substrates [5,6,7,23,24,25,32,33]. Laser processing provides another maskless route for local electrode patterning and structural modification; however, for practical on-chip integration, the resulting silicon electrode needs to retain mechanical integrity, electrical connectivity, and interfacial stability [23,24,25,26].

Printable routes trade tooling simplicity and shape freedom for active-material dilution and packing penalties. Binder, conductive carbon, rheology modifiers, residual porosity, and low dry density all reduce the silicon volume fraction, so a high silicon-specific capacity can coexist with modest footprint-normalized or volumetric device energy. Printed-electrode studies should therefore report solids composition, dry thickness and density, active-silicon fraction, and areal capacity, rather than only mAh g^−1^ normalized to silicon. This limitation makes printing most attractive for rapid prototyping, flexible substrates, and post-CMOS hybrid integration rather than for applications that require the highest packaged volumetric energy density [23,24,25,30,33].

## 8. Full-Cell Integration of Silicon Anodes in Miniaturized Batteries

### 8.1. Cathode Pairing and Capacity Balancing

Capacity balance should be expressed using usable areal capacities rather than the theoretical silicon capacity. The effective anode/cathode (N/P) ratio must account for first-cycle irreversible lithium loss: once cathode capacity and lithium inventory are limiting, excess silicon cannot raise full-cell capacity and instead adds SEI area, inactive volume, and mechanical risk. This is why high capacity measured against a lithium-metal counter electrode is an inadequate proxy for practical microbattery energy [3,32,41,85].

Figure 2b illustrates a practical full-cell implementation in which the silicon anode is integrated with the remaining battery components in a sealed microbattery architecture. High-voltage cathodes may improve energy density, but they often require high-temperature crystallization or careful electrolyte compatibility. Lower-temperature or amorphous cathodes may be easier to integrate with CMOS-compatible processes, but they can have lower capacity or rate capability. The optimum pairing depends on the thermal budget, deposition method, footprint, and intended load profile. For a complete microbattery, an excellent silicon anode is useful only if the cathode, electrolyte, current collectors, and package can be integrated with comparable reliability [2,8,9,10,11,22,78,79,80,81].

A silicon anode must be paired with a cathode that matches its capacity, voltage window, processing temperature, and deposition route [5,6,7,8,9,10,11,22,32,33]. Common cathode materials for thin-film and microbattery systems include LiCoO_2_, LiMn_2_O_4_, LiFePO_4_, V_2_O_5_, TiS_2_, and related oxide and sulfide materials [8,9,10,11,22]. Sputtered LiCoO_2_ is widely used but often requires thermal treatment to achieve high crystallinity, which can conflict with back-end-of-line thermal limits [8,9,10,11,22].

**Figure 7:**
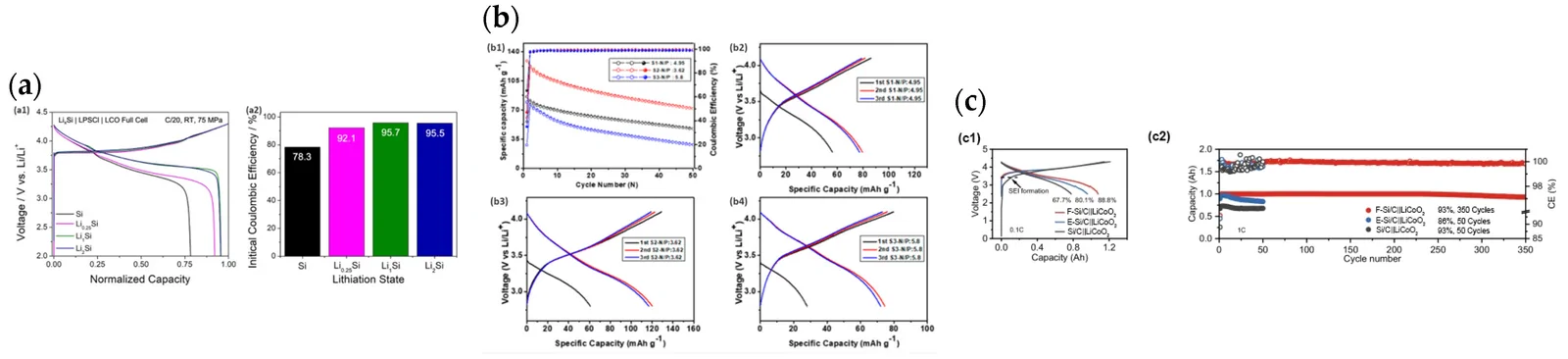
Full-cell design considerations for translating high-capacity Si anodes into practical batteries. (**a**) First-cycle voltage profiles and initial Coulombic efficiencies of Si and prelithiated LiₓSi anodes paired with an LCO cathode, demonstrating compensation of first-cycle lithium loss through controlled prelithiation [120]. (**b**) Cycling performance and initial charge–discharge profiles of Si-containing composite-anode/NCM622 full cells with different negative-to-positive capacity ratios (N/P ratios), illustrating the importance of electrode-capacity balancing for full-cell utilization and stability [121]. (**c**) First-cycle voltage profiles and cycling performance of 1.2-Ah Si/C–LiCoO_2_ pouch cells with different prelithiation treatments, demonstrating the translation of lithium-inventory control to practical full-cell operation [122]. Panels (**a**–**c**) are adapted from Refs. [120,121,122], respectively, under the terms of the Creative Commons Attribution 4.0 International License. For unambiguous reference, the two source-internal plots within panel (**a**) are designated (**a1**) and (**a2**) and show the voltage–capacity profiles and initial Coulombic efficiencies, respectively. The four source-internal plots within panel (**b**) are designated (**b1**)–(**b4**), with (**b1**) showing cycling/Coulombic-efficiency behavior and (**b2**)–(**b4**) showing representative charge–discharge profiles for different N/P ratios. The two source-internal plots within panel (**c**) are designated (**c1**) and (**c2**) and show the first-cycle voltage profile/SEI-formulation inset and long-term cycling/Coulombic-efficiency response, respectively.

Figure 7 highlights the transition from Si-anode performance measured at the electrode level to lithium-inventory-limited full-cell operation. Because the cathode provides a finite lithium inventory, the first-cycle irreversible loss of Si directly reduces the usable full-cell capacity. Controlled prelithiation can compensate for this loss and substantially increase the initial Coulombic efficiency (Figure 7a) [120]. The benefit of a high-capacity Si anode also depends critically on electrode-capacity balancing. As shown in Figure 7b, variation in the N/P ratio changes the accessible full-cell capacity, initial efficiency, and subsequent cycling behavior, demonstrating that excess anode capacity does not necessarily translate into higher usable cell energy [121]. At a more practical cell scale, prelithiation strategies can translate into improved capacity utilization and cycling performance in Ah-class Si-containing full cells (Figure 7c) [122]. These results emphasize that Si-anode performance should ultimately be evaluated using balanced full cells with explicitly reported N/P ratios, lithium inventories, areal capacities, and first-cycle efficiencies.

Two quantities should therefore be stated explicitly for silicon-containing full cells: the designed N/P ratio based on reversible areal capacity and the retained lithium inventory after formation. If the first-cycle silicon loss exceeds the lithium reserve supplied by the cathode, cell capacity is irreversibly clipped before long-term cycling begins. Prelithiation can restore inventory but adds processing, contamination, handling, and encapsulation burdens, as discussed in Section 8.2 [41,123,124,125].

### 8.2. Prelithiation

Prelithiation is particularly important for silicon because the first-cycle lithium loss can be substantial. In a half-cell, the lithium metal masks this loss; however, in a full microbattery, the lithium inventory is finite and is usually supplied by the cathode. Methods such as electrochemical prelithiation, sacrificial lithium-rich additives, direct contact with stabilized lithium sources, and deposition of lithium-containing layers can compensate for the initial loss. However, these methods must be adapted to wafer-level processing, safety requirements, and encapsulation [41,42,43,44,45,62,63,64,123,124,125].

For on-chip devices, prelithiation also raises concerns regarding contamination and handling. Exposed lithium or reactive lithiated silicon is difficult to process in a standard microfabrication environment and may require dry-room or inert-atmosphere handling. A practical strategy may be to combine moderate Si utilization, artificial interphase coatings, and controlled prelithiation rather than relying on extreme silicon loading. Reports on silicon-anode microbatteries should explicitly state whether the cell is lithium-limited, how first-cycle loss is compensated, and whether the reported capacity is realistic for a sealed full cell [3,8,9,10,11,17,18,19,20,21,22,41,42,43,44,45,123,124,125].

### 8.3. Encapsulation and Packaging

Packaging is not an afterthought in miniaturized batteries; it is often a major fraction of the final device volume. A microbattery with excellent electrode-level areal capacity can lose its advantage if the package is thick, laterally oversized, or incompatible with wafer-level processing. Liquid-electrolyte cells require hermetic sealing and leakage control, whereas solid-state cells require protection from moisture, oxygen, and mechanical damage. In both cases, the package must preserve electrical access and avoid contaminating nearby circuits [1,2,5,6,7,8,9,10,11,22,28,29,30,31,32,33].

For silicon anodes, packaging must also tolerate mechanical changes during cycling. Even if electrode expansion is locally accommodated by pillars or pores, the packaged cell may experience pressure changes, interfacial delamination, or encapsulation stress. Wafer bonding, thin-film barriers, glass caps, metal caps, and multilayer inorganic/organic barriers offer different trade-offs in terms of thickness, permeability, thermal budget, and mechanical compliance. Future studies should therefore report the packaged energy density in addition to electrode capacity and should clearly distinguish between open half-cell tests, laboratory full cells, and sealed on-chip devices [1,2,3,5,6,7,8,9,30,31,32,33,85,86,87].

## 9. Characterization and Reporting Metrics

A major obstacle in comparing silicon-anode microbattery studies is inconsistent normalization. Gravimetric capacity can be misleading because the mass of a microfabricated silicon electrode may be small relative to the substrate, current collectors, electrolyte, and package. Areal capacity is more relevant for footprint-limited devices, but even the areal capacity can be ambiguous if the normalization area excludes contact pads, interconnects, or encapsulation margins. Volumetric energy density is also sensitive to whether only active material, the electrode stack, or the fully packaged device volume is used [1,2,3,5,6,7,8,9,28,29,30,31,32,33].

Recommended reporting should include several levels of metrics: active-silicon capacity, electrode-footprint areal capacity, full-cell areal energy, stack-level volumetric energy, packaged-device volumetric energy, Coulombic efficiency, rate capability, impedance growth, and cycle retention. Structural reporting should include top-view and cross-sectional images before and after cycling, feature dimensions with statistics, coating thicknesses, and evidence of electrolyte/cathode conformality. For 3D architectures, it is particularly important to show whether the entire feature height is electrochemically utilized or whether only the top portion participates [3,4,5,6,7,8,9,10,11,12,13,14,15,16,17,18,19,20,21,22,32,33,100,101,102].

To support cross-study benchmarking, the quantitative benchmark compilation compiles representative data using a minimum parameter set: fabrication route, feature size or silicon loading, areal/specific capacity, initial Coulombic efficiency when reported, cycle number and retention, rate/current condition, electrolyte and half-/full-cell configuration, package status, maximum process temperature information when available, and CMOS/MEMS integration implications. “NR” is used when the cited source does not report a parameter; values with different normalization bases are not treated as directly interchangeable.

Characterization beyond capacity is important because structural and spectroscopic measurements can reveal stress, strain, interfacial degradation, and other failure signatures that are invisible in a simple capacity plot. Multimodal characterization should combine SEM/TEM, Raman spectroscopy, XRD, XPS, impedance spectroscopy, and mechanical modeling, where appropriate. Such data can distinguish between capacity fading caused by fracture, SEI growth, loss of electrical contact, electrolyte degradation, and cathode limitation. Without this diagnosis, a microbattery architecture may appear to fail electrochemically, whereas the root cause may actually be mechanical or process-related [3,4,16,17,18,19,20,21,46,47,48,49,50,51,68,100,101,102].

Because microbatteries are footprint-limited, device-level areal and volumetric metrics should be reported alongside conventional electrochemical performance metrics [1,2,5,6,7,8,9,28,29,30,31,32,33]. The active-material gravimetric capacity alone is insufficient because it can obscure the mass and volume of current collectors, electrolytes, substrates, encapsulation, and packaging [1,2,5,6,7,8,9,28,29,30,31,32,33].

Mechanical and structural characterization are essential for silicon anodes [37,38,39,40,41,42,43,44,45,46,47,48,49,50,51,52,53,54,55]. SEM and TEM can reveal cracking, pulverization, nanowire collapse, pore closure, SEI accumulation, and delamination after cycling [37,38,39,40,41,42,43,44,45,46,47,48,49,50,51,52,53,54,55]. Wafer-curvature stress measurements, nanoindentation, operando microscopy, and finite-element modeling can link geometry to stress evolution and failure probability [46,47,48,49,50,51,52,53,54,55,100,101,102].

For on-chip batteries, system-level testing is also important [1,2,28,29,30,31]. The battery should be evaluated under realistic loads, pulse profiles, temperature conditions, and packaging constraints relevant to the target microsystem [1,2,5,6,7,8,9,28,29,30,31,32,33]. Powering sensors, communication circuits, memory elements, and MEMS actuators can provide stronger evidence of practical utility than isolated half-cell tests alone [1,2,5,6,7,8,9,28,29,30,31,32,33].

## 10. Challenges and Future Directions

The most important future direction is the development of integrated process flows rather than isolated electrode demonstrations. A silicon-anode microbattery must combine silicon patterning, current collection, interface stabilization, electrolyte deposition, cathode formation, prelithiation or lithium management, and encapsulation into a sequence that preserves both electrochemical performance and wafer-level yield. Each step can be optimized individually, but the final device will be limited by the least compatible step. This is why process compatibility and manufacturability should be treated as scientific performance metrics, not merely as engineering details [1,2,3,6,7,8,9,30,31,80,81,82,85,86,87].

A second direction is the creation of design maps that connect geometry to performance. Instead of reporting a single optimized pillar height or nanowire length, future studies should map diameter, pitch, height, porosity, coating thickness, and cathode balance to areal capacity, rate capability, Coulombic efficiency, and cycle life. Such maps would allow researchers to identify regimes where increasing surface area is beneficial and regimes where interface loss dominates. Combined with finite-element and electrochemical transport modeling, these maps could support computer-aided design of silicon-anode microbatteries [17,18,75,76,77,100,101,102].

Finally, the field needs more sealed, application-level demonstrations. A microbattery intended for an autonomous sensor should be tested under pulse profiles relevant to sensing and communication, while an implantable or wearable device should be evaluated under appropriate temperature, bending, and packaging conditions. Demonstrations that power a microsystem, survive storage, and maintain performance after packaging will be more convincing than half-cell data alone. Silicon provides a unique opportunity because it is both an active material and the dominant platform for microfabrication; realizing that opportunity will require co-design across materials chemistry, mechanics, lithography, and packaging [1,2,3,5,6,7,8,9,28,29,30,31,32,33].

**Figure 8:**
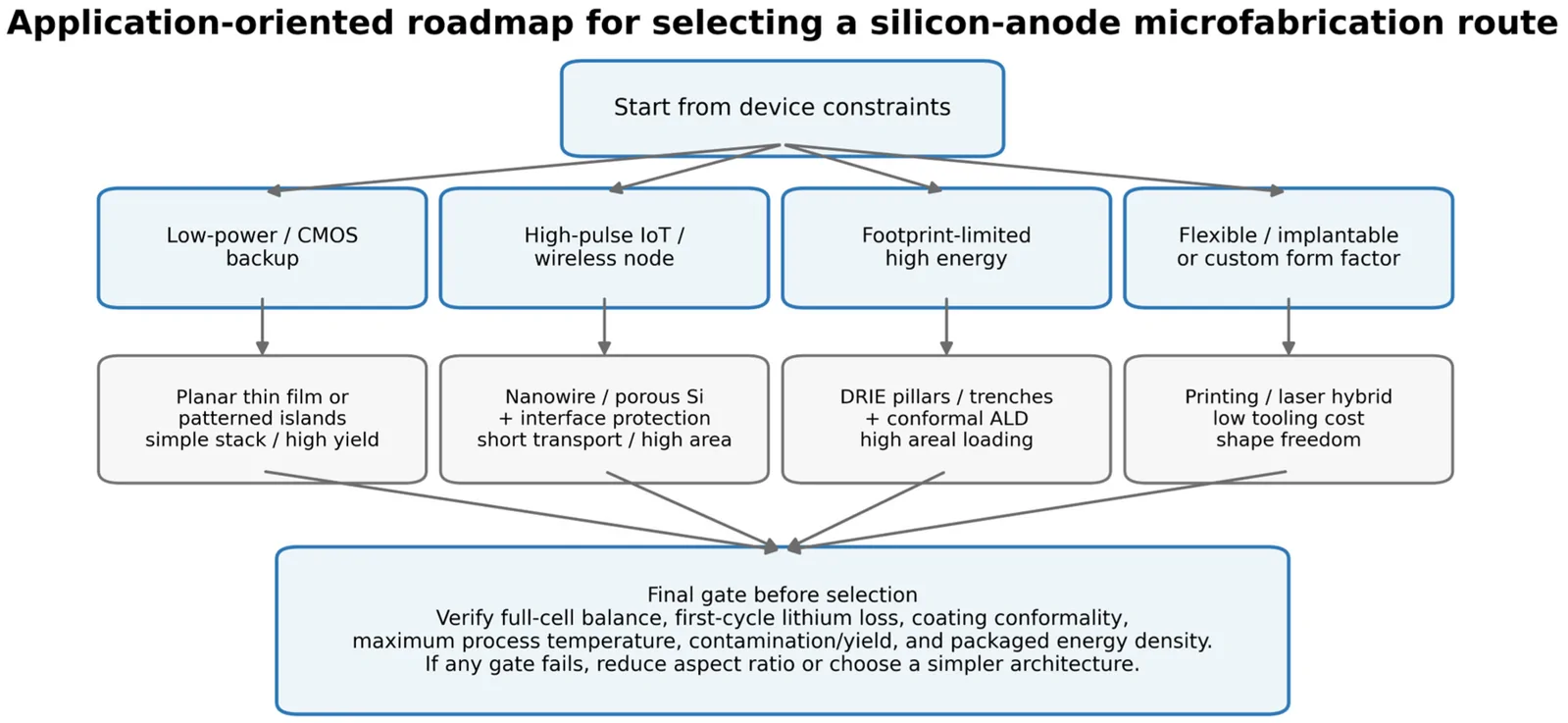
Application-oriented design roadmap for selecting a silicon-anode microfabrication strategy. The pathway links device-level requirements to candidate fabrication routes and applies a final integration gate based on full-cell balance, lithium inventory, conformality, thermal budget, contamination and yield, and packaged energy density. Original schematic prepared by the authors from the synthesis developed in this review. Arrows indicate the direction of the selection workflow from application requirements to candidate fabrication routes and then to the final integration gate.

Architecture selection should therefore begin from the application rather than from the maximum achievable silicon surface area. Low-power CMOS backup favors planar films or patterned islands because process simplicity, yield, and leakage control dominate; high-pulse wireless nodes can justify nanowire or porous-silicon architectures when interface protection maintains impedance; footprint-limited high-energy devices can justify DRIE pillars or trenches only when the electrolyte/cathode stack can be deposited conformally; printing or laser-hybrid routes are most compelling for flexible, custom, or post-CMOS devices. In every case, the final gate is full-cell balance, first-cycle lithium loss, process-temperature/contamination limits, sealing, yield, and packaged energy density. Figure 8 summarizes this decision pathway.

## 11. Conclusions

Overall, microfabricated silicon anodes should be understood as engineered systems rather than as simple high-capacity materials. Their performance depends on how silicon geometry accommodates expansion, how interfaces suppress irreversible reactions, how current collectors preserve electronic pathways, and how the complete cell is packaged. The review therefore emphasizes that microfabrication is not only a method for making small electrodes; it is a tool for controlling the mechanical, interfacial, and transport boundary conditions that determine battery lifetime. This perspective is especially important for on-chip and miniaturized batteries, where the electrode footprint, inactive volume, and process sequence strongly influence practical energy density.

The most promising path forward seems to combine deterministic silicon patterning with conformal interface engineering and realistic full-cell design. Planar films will remain useful for simple solid-state stacks and mechanistic studies; patterned islands and pillars will provide mechanically robust intermediate architectures; nanowires and porous silicon will offer high surface area when paired with effective interphase control; and hybrid printing or laser-based routes may enable application-specific layouts. By comparing these strategies with common metrics and by reporting packaged-device performance, the field can move from impressive silicon half-cell demonstrations toward manufacturable microscale power sources.

The practical selection rule is application-first: use the simplest architecture that meets the required areal energy and pulse-power targets while remaining compatible with lithium inventory, maximum process temperature, contamination control, sealing, and wafer-level yield requirements. A more complex 3D silicon structure is justified only when its package-level energy or power gain outweighs the additional interfacial and manufacturing risk. The summary tables together with Figure 8 provide a compact decision framework for making this trade-off.

Table 1 summarizes the principal microfabrication strategies for silicon anodes in miniaturized batteries and their main advantages, limitations, and target battery formats.

Table 2 compares representative process–structure–performance relationships and the reporting metrics most relevant to on-chip silicon-anode batteries.

Table 3 summarizes the key silicon-anode design parameters, their positive and negative effects, and practical optimization directions.

Table 4 positions the present review relative to representative related reviews in terms of silicon specificity, microfabrication focus, on-chip integration, and full-cell/packaging coverage.

Table 5 compiles representative quantitative electrochemical and integration benchmarks for silicon-anode microbattery strategies.

## Figures and Tables

**Table 1:** Microfabrication strategies for silicon anodes in miniaturized batteries.

Strategy	Typical Process	Main Advantage	Main Limitation	Suitable Battery Type
Planar Si thin film [88,89,90]	Sputtering, evaporation, PECVD, LPCVD	Simple, wafer-scale, solid-state compatible	Low areal capacity; stress in thick films	Thin-film microbattery
Patterned Si islands [46,48,113]	Lithography plus etching or lift-off	Reduced crack propagation; controlled footprint	Lower packing density	Planar on-chip cells
Si micropillars [2,6,7,8,9,12]	Photolithography plus DRIE	High surface area; direct substrate contact	Difficult conformal coating at high aspect ratio	3D microbattery
Si microtrenches [6,7,8,9,12]	DRIE or anisotropic wet etching	High loading; vertical surface area	Electrolyte and cathode filling challenge	3D solid-state or gel cells
Si nanowires [4,35,36,67]	MACE, cryogenic DRIE, CVD	Stress relaxation; short diffusion length	High SEI area; fragile structures	High-rate microanodes
Porous/black Si [13,14,15,57,103,104,105]	MACE, plasma etching, laser texturing	Large surface area; internal free volume	Large irreversible lithium loss	Liquid or gel electrolyte cells
Coated Si scaffold [48,49,50,51,57,108,111,112]	ALD, CVD, sputtering	Interface stabilization; conformal protection	Added inactive mass; process time	Solid-state microbatteries
Printed Si composite [20,21,30,33]	Inkjet, aerosol jet, screen printing	Low-cost; customizable geometry	Binder and carbon reduce volumetric density	Flexible or custom microcells
Hybrid lithography-printing [1,5,30,33]	Lithography plus printing or filling	Combines precision and active loading	Multistep complexity	Practical miniaturized cells

**Table 2:** Representative process–structure–performance relationships for silicon anodes in on-chip batteries.

Process Family	Representative Silicon Structure	Key Performance-Relevant Variable	Main Integration Issue	Recommended Reporting Metric
Planar PVD/CVD Si [16,88,89,90]	Amorphous or crystalline Si film	Film thickness, adhesion, residual stress	Cracking and delamination in thicker films	Areal capacity, stress evolution, post-cycling cracks
Lithographic patterning [1,46,48,113]	Islands, stripes, microdisks	Island width, pitch, current-collector layout	Loss of packing density with excessive spacing	Crack density, active-area fraction, cycle retention
DRIE/cryogenic RIE [4,56,91,92,93,95]	Micropillars, trenches, vertical nanowires	Aspect ratio, sidewall angle, mask stability	Conformal coating and etch-profile defects	Height statistics, coating coverage, capacity per footprint
MACE/porous Si [13,14,15,103,104,105]	Porous silicon, black silicon, Si nanowires	Pore size, catalyst residue, open porosity	SEI growth and first-cycle loss	ICE, pore accessibility, catalyst removal evidence
ALD/interface coating [48,49,50,51,111,112]	Coated Si scaffold or nanowire array	Coating chemistry and thickness	Throughput and conformality in dense structures	Impedance growth, Coulombic efficiency, coating integrity
Full-cell integration [7,8,9,31,58,59,60,61,80,81,82,85,86,87]	Si anode paired with thin-film cathode	Cathode/anode balance and lithium inventory	Prelithiation and package compatibility	Full-cell areal energy and packaged volumetric energy

**Table 3:** Key silicon-anode design parameters, associated trade-offs, and recommended optimization directions.

Design Parameter	Positive Effect	Negative Effect	Optimization Direction
Si thickness [16,46,68,88,89,90]	Higher areal capacity	Higher stress and fracture risk	Use thin films, islands, or 3D geometry
Surface area [24,35,36,37,38,39,40,41,42,43,44,45]	Faster kinetics and higher utilization	More SEI formation	Use artificial SEI and coatings
Porosity/spacing [13,14,15,37,38,39,40,41,42,43,44,45,53]	Accommodates expansion	Reduces active packing density	Balance void volume and loading
Aspect ratio [4,12,17,18,56,76,77]	Higher footprint capacity	Coating and filling difficulty	Match geometry to ALD or infiltration process
Conductive coating [40,41,52,57,108]	Improves electron transport	Adds inactive mass	Use ultrathin conformal layers
Solid electrolyte [7,8,9,58,59,60,61,77,80,81,86,87]	Improves safety and integrability	Can be brittle and resistive	Use compliant or artificial interlayers
Low-temperature process [1,22,31,57,58,59,60,61,79]	Improves CMOS compatibility	May reduce film crystallinity	Develop amorphous or nanostructured materials
Wafer-level process [1,2,3,4,6,7,8,9,12,13,14,15,16,17,18,19,20,21,22,30,31]	Improves scalability	Requires uniformity and yield control	Standardize etch and deposition windows

**Table 4:** Positioning of the present review relative to representative related reviews.

Review	Silicon-Specific Anode Focus	Microfabrication-Route Focus	On-Chip/CMOS-MEMS Integration	Full-Cell/Packaging Focus	Primary Emphasis/Gap Relative to This Review
Ref. [1] (2025)	No; multi-chemistry microbatteries	Strong; photolithography-centered	Strong on-chip patterning; semiconductor-process compatibility discussed	Partial	Photolithography as the organizing axis; less silicon-specific mechanics/full-cell manufacturing analysis
Ref. [2] (2024)	Yes	Strong; broad 3D Si routes/architectures	On-chip 3D integration discussed	Strong at architecture level	Comprehensive 3D Si micro-LIB review; less explicit process-to-manufacturability decision framework
Ref. [3] (2022)	Partial; multi-chemistry	Broad micro-LIB fabrication	Strong on-chip focus	Broad cell-level discussion	On-chip micro-LIB landscape across chemistries rather than silicon-specific process/mechanics
Refs. [24,41]	Yes	Nanostructure/material strategy rather than wafer integration	Limited	Limited	Silicon materials, size/geometry, and failure mechanisms; not focused on microbattery process integration
Present review	Yes	Planar film, lithography, DRIE, MACE, NIL, ALD, printing, packaging	Explicit CMOS/MEMS process and yield assessment	Explicit balancing, lithium inventory, prelithiation, packaging	Unified process-structure-performance-integration framework with quantitative benchmarks and application-oriented selection roadmap

**Table 5:** Representative quantitative electrochemical and integration benchmarks for silicon-anode microbattery strategies.

Study/Route	Geometry/Loading	Capacity/ICE	Cycling/Rate	Electrolyte and Cell	Package/Process Information	Integration Interpretation
Ref. [90] planar vacuum-deposited Si	50 nm Si film on 30 μm Ni foil	>3500 mAh g^−1^; initial loss reported, ICE NR	Maintained for 200 cycles at 2C	PC + 1 M LiClO_4_; Li half cell	Unpackaged; vacuum deposition	High specific capacity at very low loading; useful planar baseline, but device areal capacity is limited
Ref. [57] ICP-etched black-Si + Cu	Nanostructured Si; 50 nm Cu coating	632.7 μAh cm^−2^ first discharge; ICE 47.9%	~70% capacity retained after 20 cycles at 50 μA cm^−2^	Liquid electrolyte; Li half cell, 0.1–2.0 V	Unpackaged; ICP etch + sputtering; CMOS-compatible process claimed	Shows manufacturable Si nanostructuring but also large first-cycle lithium loss
Ref. [4] cryogenic ICP-RIE Si nanowires	Vertical Si NW array tested at ~1 μm diameter × ~3.9 μm height (AR ~3.9); fabrication study achieved AR up to 22	Prelithiation discharge/charge: 0.497/0.463 mAh cm^−2^; ICE 93.07%; post-prelithiation discharge 0.30 → 0.25 mAh cm^−2^	16.67% discharge-capacity fade through 20 cycles at 0.06 mA cm^−2^	Li half cell; liquid electrolyte	Unpackaged; cryogenic ICP-RIE; mask/process dependent	Deterministic high-AR geometry, but cycling and full-stack conformality remain bottlenecks
Ref. [85] monocrystalline Si microbattery	Wafer-grade Si anode/housing; 4 × 4 mm class device	Up to ~10 mAh cm^−2^ class discharge density; CE >99.9% for first 180 cycles in reported configuration	>100 full cycles; ~17% fade after 200 cycles for a 0.5 mAh cell; peak power up to ~200 mW cm^−2^	Rechargeable full microbattery	Sealed device; semiconductor-grade monocrystalline Si and wafer-compatible construction	Demonstrates why packaged full-cell metrics can be more informative than anode half-cell capacity
Ref. [81] low-temperature all-solid-state thin-film microbattery	Silicon-based thin-film microbattery cell	Areal capacity up to 188 μAh cm^−2^; ICE NR	High-rate operation at 34.4 mA cm^−2^; 10^6^ fast charge–discharge cycles at 20 mA cm^−2^	All-solid-state full microbattery	Developed at low temperature for on-chip integration	Directly targets the electrochemistry-microelectronics integration barrier
Ref. [126] CVD Si nanowires	SiNW film grown at 540 °C	~1200 mAh g^−1^ at C/100; ~900 mAh g^−1^ at C/10; ~600 mAh g^−1^ at C/4	Capacity recovered near 1200 mAh g^−1^ on returning to C/100; stable ~500 mAh g^−1^ for ≥50 cycles with restricted cutoff	Li half cell; liquid electrolyte	Unpackaged; CVD at 540 °C	Good rate response, but growth temperature is a major back-end CMOS constraint

Note: NR = not reported in the cited source. Cross-study values use different normalization bases and test conditions and should therefore be interpreted as representative benchmarks rather than as a strict ranking.

## Data Availability

No new data were created or analyzed in this review. Data sharing is not applicable to this article.
